# National-scale remotely sensed lake trophic state from 1984 through 2020

**DOI:** 10.1038/s41597-024-02921-0

**Published:** 2024-01-16

**Authors:** Michael F. Meyer, Simon N. Topp, Tyler V. King, Robert Ladwig, Rachel M. Pilla, Hilary A. Dugan, Jack R. Eggleston, Stephanie E. Hampton, Dina M. Leech, Isabella A. Oleksy, Jesse C. Ross, Matthew R. V. Ross, R. Iestyn Woolway, Xiao Yang, Matthew R. Brousil, Kate C. Fickas, Julie C. Padowski, Amina I. Pollard, Jianning Ren, Jacob A. Zwart

**Affiliations:** 1 U.S. Geological Survey, Madison, WI USA; 2 University of Wisconsin – Madison, Madison, WI USA; 3 U.S. Geological Survey, Carrboro, NC USA; 4 U.S. Geological Survey, Boise, ID USA; 5 Oak Ridge National Laboratory, Oak Ridge, TN USA; 6 U.S. Geological Survey, Kearneysville, WV USA; 7 Carnegie Institution for Science, Pasadena, CA USA; 8 Longwood University, Farmville, VA USA; 9 University of Wyoming, Laramie, WY USA; 10 U.S. Geological Survey, Los Angeles, CA USA; 11 Colorado State University, Fort Collins, CO USA; 12 Bangor University, Menai Bridge, Anglesey UK; 13 Southern Methodist University, Dallas, TX USA; 14 U.S. Geological Survey, Sioux Falls, SD USA; 15 University of California - Santa Barbara, Santa Barbara, CA USA; 16 Washington State University, Pullman, WA USA; 17 U.S. Environmental Protection Agency, Washington, DC USA; 18 University of Nevada - Reno, Reno, NV USA; 19 U.S. Geological Survey, Pittsburgh, PA USA

**Keywords:** Limnology, Ecosystem ecology, Freshwater ecology, Environmental sciences

## Abstract

Lake trophic state is a key ecosystem property that integrates a lake’s physical, chemical, and biological processes. Despite the importance of trophic state as a gauge of lake water quality, standardized and machine-readable observations are uncommon. Remote sensing presents an opportunity to detect and analyze lake trophic state with reproducible, robust methods across time and space. We used Landsat surface reflectance data to create the first compendium of annual lake trophic state for 55,662 lakes of at least 10 ha in area throughout the contiguous United States from 1984 through 2020. The dataset was constructed with FAIR data principles (Findable, Accessible, Interoperable, and Reproducible) in mind, where data are publicly available, relational keys from parent datasets are retained, and all data wrangling and modeling routines are scripted for future reuse. Together, this resource offers critical data to address basic and applied research questions about lake water quality at a suite of spatial and temporal scales.

## Background & Summary

Lakes and reservoirs are of critical importance to society, directly providing drinking water and supporting food production, sanitation, and transportation. Millions of people worldwide face intermittent clean water availability due to climatic and anthropogenic stressors^1^. Current literature suggests that changes in surface water quantity and quality are highly heterogeneous, and trends globally suggest that factors such as ice cover, air temperature, humidity, and lake surface area are likely interacting regionally to affect freshwater ecosystems in synergistic ways^2–7^. To gain a better understanding of the potential threats to freshwater ecosystems, new technologies must be engaged. Satellite-based Earth observations (hereafter “remote sensing”) are particularly useful as they can provide information at spatial and temporal scales that are currently impossible to replicate via ground-based observations.

Although remote sensing’s usefulness to track changes in water quantity has enabled analyses of water availability from local-to-global scales^8–11^, investigations of water quality have historically been more limited in scale and scope. However, remote sensing now offers powerful approaches to assessing patterns and trends in water quality^2,12–15^, and data harmonization efforts allow for greater interoperability between *in situ* collections and remote sensing imagery^16,17^. Among studies of remotely sensed metrics of water quality, the majority have centered around specific constituents, such as secchi disk depth, chlorophyll, or suspended sediment, without necessarily offering holistic metrics of ecosystem productivity.

**Fig. 1:**
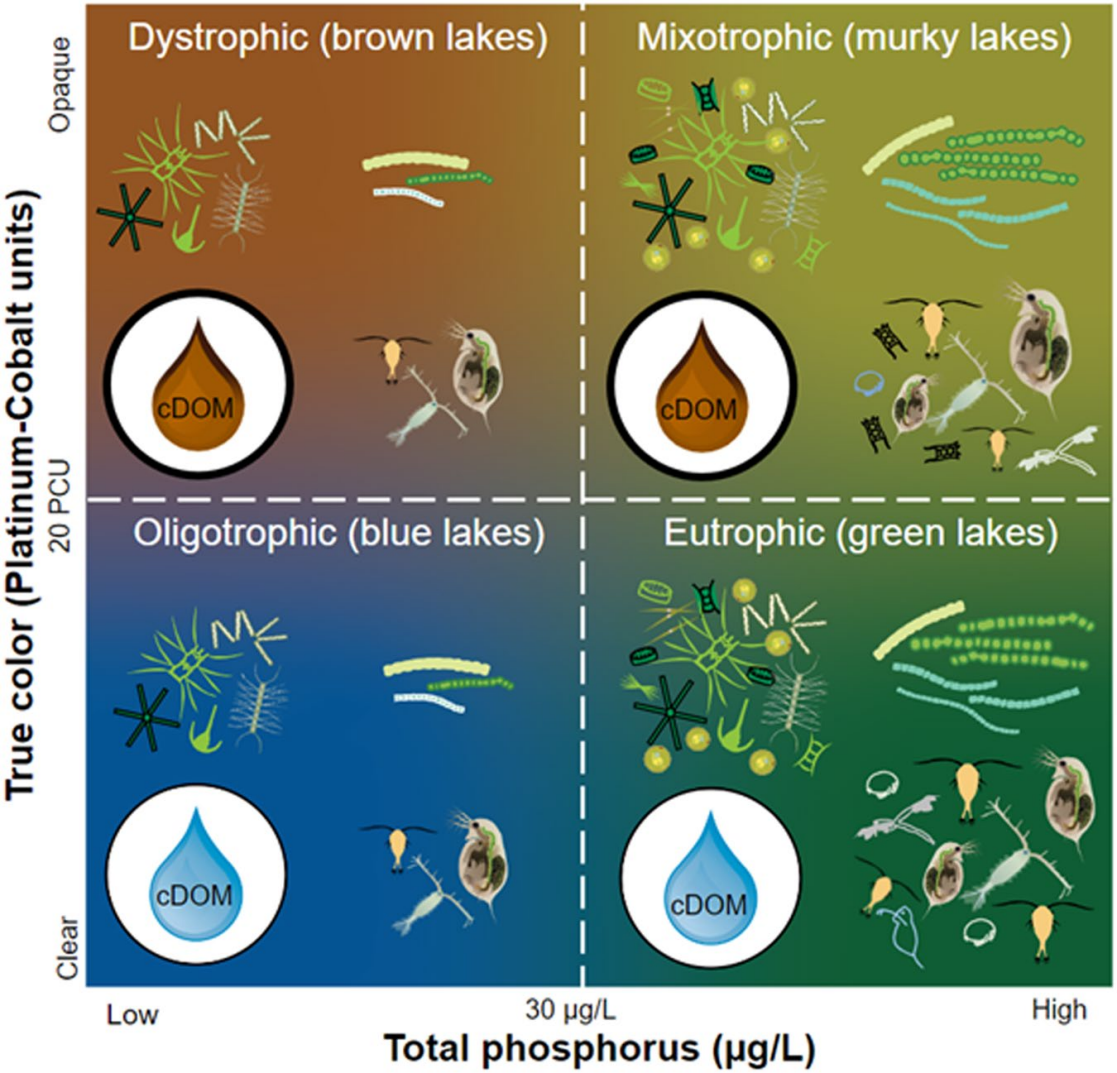
Nutrient-Color Paradigm (NCP) scheme for classifying oligotrophic, eutrophic, dystrophic, and mixotrophic lakes. Schematic is adapted from Williamson *et al*.^22^ and Webster *et al*.^23^, and characteristic lake descriptions broadly stem from results presented in Leech *et al*.^24^ and Oleksy *et al*.^92^ Within each NCP-quadrant, there are a suite of physical, chemical, and biological characteristics that distinguish each type of lake: colored Dissolved Organic Matter (cDOM), primary production, cyanobacterial abundance, and higher order production. Lower cDOM concentrations (blue water drops) are characteristic in oligotrophic and eutrophic lakes. When cDOM is low, light can transmit through the water column more deeply, allowing for primary producers to undergo photosynthesis and zooplankton to consume primary producers (oligotrophic). When nutrients, such as phosphorus, are at higher concentrations and cDOM is low (eutrophic), primary production, cyanobacterial abundance, and higher order production can all increase, resulting in increased biomass. When cDOM concentrations are high (brown water drop) and nutrient concentrations are low (dystrophic), the increased light attenuation can result in decreased primary production, which can reciprocally cause decreased higher order production. Lastly, when nutrient and cDOM concentrations are both high (mixotrophic), primary production, cyanobacterial abundance, and higher order production can exceed values observed when solely cDOM or nutrient concentrations alone are higher. Phytoplankton and filled-in zooplankton cartoons were downloaded from the University of Maryland Center for Environmental Science Integration and Application Network (https://ian.umces.edu/media-library/). Phytoplankton were designed by Tracey Saxby of the Integration and Application Network, Dieter Tracey of the Water and Rivers Commission, Kim Kraeer and Lucy Van Essen-Fishman of the Integration and Application Network. Transparent crustacean zooplankton and rotifer cartoons were drawn by Stephanie E. Hampton.

Lake trophic state (LTS) is an example of a metric intended to provide holistic assessments of a lake’s aggregate physical (e.g., light attenuation), chemical (e.g., nutrient concentrations), and biological processes (e.g., productivity). Broadly speaking, LTS is a property closely associated with a lake’s characteristic autochthonous and allochthonous productivity as well as water color^18^. Eutrophic lakes are green, oligotrophic lakes are blue, and dystrophic lakes are brown (Fig. 1). From color-trophic state connections, fundamental limnological principles center around linking trophic states to characteristic properties (Fig. 1). For example, oligotrophic lakes are usually characterized by having lower phosphorus concentrations, low offshore but comparably higher nearshore productivity, and low colored dissolved organic matter (Fig. 1). In contrast, eutrophic lakes have higher phosphorus concentrations and higher phytoplankton biomass (Fig. 1).

In a management context, the language of LTS has historically been used to describe conditions relative to nutrient enrichment. For example, following the 1971 announcement of US Federal efforts to limit the use of phosphorus in detergents, the U.S. Environmental Protection Agency (U.S. EPA) and state water resource management agencies launched a National Eutrophication Survey^19^. The survey assessed trophic state, defined as nutrient enrichment, of lakes influenced by wastewater treatment plants. In this case, LTS language was used to focus on and communicate about eutrophication, whereas dystrophication aspects of the framework were not as prominent. These language patterns likely carry over to contemporary uses. Because discussions may have focused on eutrophication in the past, modern tools and frameworks could be enhanced by remotely sensed water quality data that capture aspects of both eutrophication and dystrophication. For example, as climate changes, drinking water utility managers will increasingly face compounding hazards that could negatively impact lakes and reservoirs that supply hundreds of millions of people with drinking water^20^. Data and tools that provide remotely sensed information on LTS could improve the ability to observe multidecadal changes in water quality and save resources by better targeting field monitoring.

Although LTS is often employed as a classification system for characterizing autotrophic production^21^, the Nutrient-Color Paradigm (NCP) is an empirically tested framework for discriminating LTS based off two variables: (1) phosphorus concentrations, a proxy for nutrient availability and primary productivity; and (2) colored dissolved organic matter or turbidity measured in platinum-cobalt units, both proxies for water transparency^22–24^. By combining characteristic metrics of a lake’s primary productivity and optical properties, the NCP presents a powerful system for discriminating LTS, where both autochthonous and allochthonous processes are considered. Leveraging the relationship between LTS, nutrient concentrations, and water clarity, it is possible to transform remotely sensed lake surface reflectance observations into meaningful limnological and ecosystem properties.

Here, we present the first national-scale compendium of LTS that has been built from remotely sensed lake color (i.e., red, green, blue, and near-infrared surface reflectances). The dataset, referred to as LTS-US, is derived from (1) coordinated, continental-scale *in situ* measurements, where LTS has been documented for select lakes and years, and (2) characteristic Landsat surface reflectance values for each lake’s Chebyshev center (the point in a polygon furthest from the edge). Using *in situ* LTS, we can build predictive models to associate LTS with characteristic reflectance values, and then apply predictive models to lakes with unknown trophic states. Together, the dataset contains predictions for 55,662 lakes of at least 10 ha in area with annual estimates of LTS from 1984 through 2020. By coupling satellite-based remote sensing with fundamental limnological principles, the LTS-US dataset provides the means to apply the NCP at the national scale to identify macroscale patterns and trends in LTS. Further, this approach moves beyond remote sensing of individual parameters to provide insights into lakes’ physical, chemical, biological, and ecosystem properties.

## Methods

**Fig. 2:**
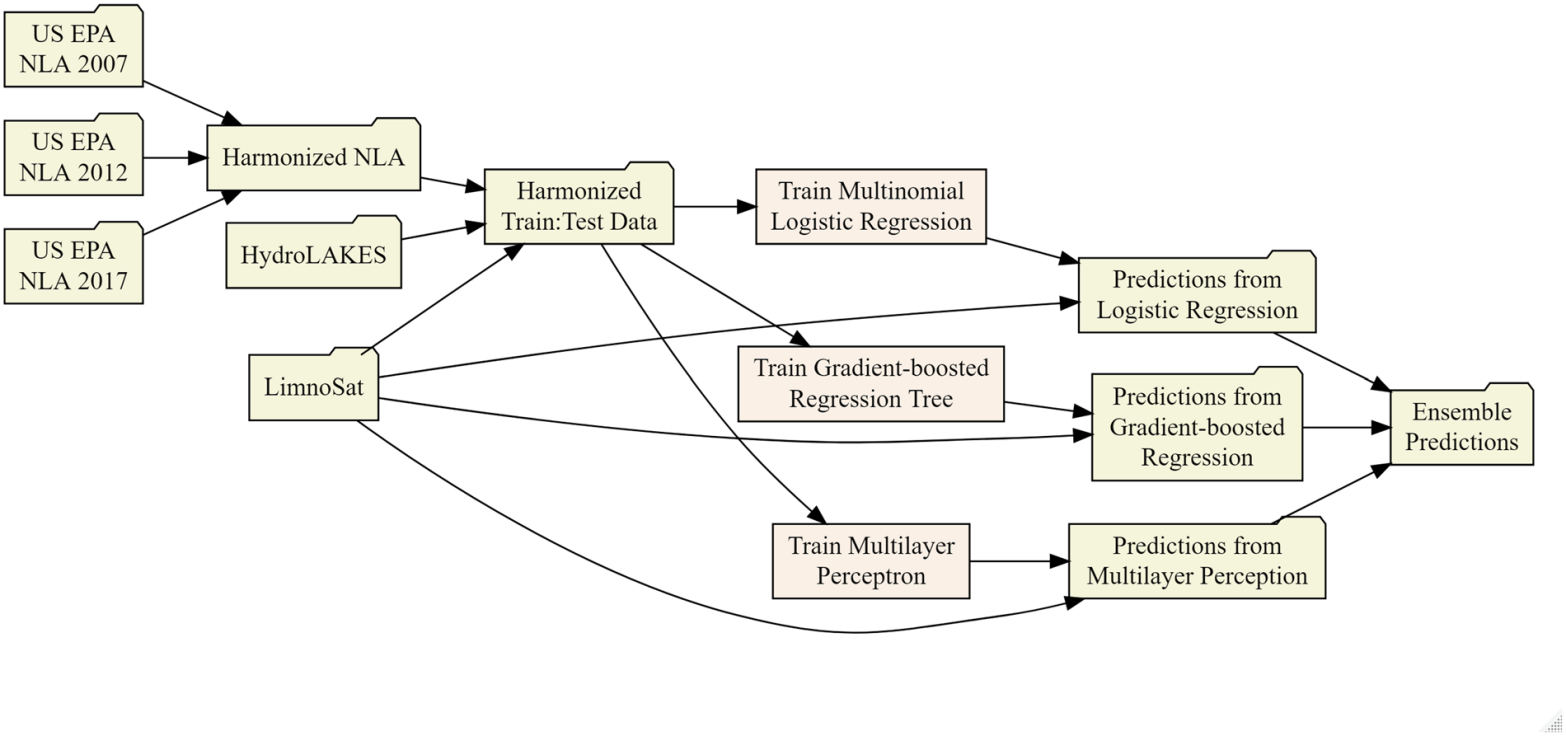
Flowchart for data harmonization, modeling, and prediction steps of the LTS-US dataset pipeline. Steps shaped as a file-folder correspond to an intermediary data product, and rectangles correspond to an intermediary model. Data aggregation combines data from the U.S. EPA’s National Lakes Assessment, HydroLAKES, and LimnoSat-US to create a single, harmonized dataset of *in situ* lake trophic states with paired remotely sensed surface reflectances. Model training steps create multinomial logistic regression, multilayer perceptron, and extreme gradient boosted regression tree models. Each fitted model is then applied to the entire LimnoSat-US data, where national-scale predictions are made for each modeling method. Probabilistic predictions are then averaged to create ensemble predictions of lake trophic state. Quality control steps (described in “Technical Validation”) use both the ensemble and individual model predictions to assess model performance. Each of these four components correspond to a piece of the overall data production pipeline: data aggregation functions are described in “1_aggregate”; model training functions are described in “2_train”; national-scale prediction functions are described in “3_predict”; quality control procedures are described in “4_qc”. Flowchart was designed with the “DiagrammeR” package^93^.

The LTS-US dataset is constructed using a four-part pipeline, as shown in Fig. 2: (1) aggregate training data, (2) create classification models, (3) apply predictions to lakes outside of the training data, and (4) assess model performance and prediction validity. Individual steps within the pipeline are described below.

### Step 1: Identify Parent Datasets

#### U.S. Environmental Protection Agency National Lakes Assessment

*In situ* measurements of total phosphorus and true color were compiled from the U.S. EPA’s National Lakes Assessment (NLA)^25–29^, a synoptic sampling campaign of lakes, ponds, and reservoirs, hereafter collectively referred to as “lakes”, conducted in the contiguous U.S. every five years. Lakes used in this analysis were sampled in the summer (June-September) of 2007 (n = 1,028), 2012 (n = 1,038), or 2017 (n = 1,005). Lakes were selected from the National Hydrography Dataset (NHD, https://www.usgs.gov/national-hydrography/national-hydrography-dataset) using a randomized design stratified on aggregated Omernik level III ecoregion^30^ and lake surface area. The minimum surface area for inclusion in the 2007 assessment was 4 ha but owing to increasing resolution in the NHD was reduced to 1 ha for the 2012 and 2017 assessments. Natural lakes and reservoirs were treated equally in the site selection process.

To inform internal quality assurance within a campaign, 10% of the lakes were sampled twice within a field season. Approximately 25% of lakes were targeted for resampling in multiple years to examine temporal change. State, Tribal, Federal, and contractor field crews evaluated lakes on site to ensure that selected lakes met criteria for inclusion in the field campaign (e.g., lake ≥1 m deep). A wide set of measurements were collected at each sampled lake, but we only provide details on the variables used in this analysis. Additional details, protocols, and data are available online (https://www.epa.gov/national-aquatic-resource-surveys/nla).

Total phosphorus and true color were collected and processed in the 2007, 2012, and 2017 field campaigns^25,26,28^. In natural lakes, field crews sampled in a deep area of the lake regardless of whether the sample location was in the geometric center of the system. In reservoirs, field crews were asked to find a midpoint in the reservoir that was reasonably lentic, deep, and away from a dam. In lakes and reservoirs deeper than 50 m, field crews sampled from a location with a maximum depth of 50 m. Water was collected from 0–2 m using a vertical integrated water sampler. In lakes where the photic zone (2x Secchi depth) was <2 m, sampling was limited to the photic zone to prevent sampling of hypolimnetic water. All water samples were placed on ice and shipped overnight to the Willamette Research Station in Corvallis, Oregon for analysis. True color was estimated by visual comparison of filtered water samples to a calibrated glass color disk^31^. Total phosphorus concentrations were measured with manual alkaline persulfate digestion, followed by automated colorimetric analysis (ammonium molybdate and antimony potassium tartrate under acidic conditions, with absorbance at 880 nm) using a flow injection analyzer following standard method 4500-P-E^32^. Detailed descriptions of all water quality analyses are available in the NLA Laboratory Operations Manuals^25,27,29^.

#### HydroLAKES

HydroLAKES (v1.0)^33^ is a compendium of more than 1.4 million lake and reservoir shapefiles globally, with surface area of at least 10 ha. For an individual waterbody, HydroLAKES contains its spatial extent and location (using georeferenced polygons), a unique identifier (ranging from 1 to 1,427,688), and its morphological (area, mean depth, elevation, shoreline length etc.), hydrological (e.g., residence time, discharge, and watershed area), and geographical (e.g., name, country, continent) properties. HydroLAKES is a compilation of existing lake databases, with sources from government agencies (e.g., Natural Resources Canada, U.S. Geological Survey, European Environment Agency) and from remote sensing studies (for example, Shuttle Radar Topographic Mission Water Body Data, Global Lakes and Wetlands Database, and Global Reservoir and Dam Database). Most of the lake polygons are sourced from the Shuttle Radar Topographic Mission Water Body Data for regions between 60°S and 60°N^34^, supplemented by other datasets for higher latitudes and for underrepresented regions. More detailed information on the creation and validation of the HydroLAKES dataset can be found in Messager *et al*.^33^.

#### LimnoSat

The LimnoSat-US^35^ dataset comprises over 22 million remotely sensed observations of lake surface reflectance from 1984 to 2020. Observations cover 55,662 lakes greater than 10 ha^33^ aggregated from Landsat 5, 7, and 8 Collection 1 imagery. Each observation was calculated by taking the median surface reflectance within 120 meters of each lake’s Chebyshev center, defined as the point farthest from shore and usually located at the lake’s deepest point^36^. While many valid choices of buffer distance exist, LimnoSat-US employed a 120 m buffer to capture reflectances from a maximum of 64 Landsat pixels, which should prevent the values of a few pixels from influencing the mean. Further, extracting reflectance values from the Chebyshev center minimizes signals due to bottom reflectance and adjacent land pixels. For each Landsat observation, non-high confidence water pixels were masked using the Dynamic Surface Water Extent algorithm^37^. Observations were removed if the scene cloud cover was greater than 75%, any snow, ice, cloud, cloud shadow^38^, or hillshadow was detected over the lake’s Chebyshev center, or if there were fewer than eight high confidence water pixels within the 120 meter buffer of the lake’s Chebyshev center. For certain lakes, these filters lead to extended periods (i.e., months to years) with limited observations (see Fig. 2 in Topp *et al*.^2^). Data in LimnoSat-US are presented in a tabular format, where each row reflects a Landsat overpass for a given waterbody, and columns include median Collection 1 surface reflectance values by band extracted from pixels within 120 m of the Chebyshev center, scene-wide cloud cover, date of imagery acquisition, and number of water pixels within 120 m of the Chebyshev center.

### Step 2: Define lake trophic state

Many lakes across the United States are experiencing simultaneous changes in their water clarity, with some lakes getting greener due to eutrophication, and others getting browner from increasing terrestrially-derived organic matter, and some are simultaneously ‘greening’ and ‘browning’^24^. Given the need to discriminate between lakes that may be browning and/or greening, the Nutrient Color Paradigm (NCP) is a useful tool to assign LTS based on a lake’s characteristic color.

The NCP was initially proposed in the early 20th century, emphasizing that both autochthonous and allochthonous processes are important to understanding LTS^39–41^. Specifically, water color often affects algal biomass and light transparency independent of nutrient availability. Rodhe^42^ first assembled the four quadrants of the NCP, placing autochthony on the horizontal axis and allochthony on the vertical axis. This second dimension distinguishes “oligotrophic” (low nutrient, low color) and “eutrophic” (high nutrient, low color) lakes from “dystrophic” (low nutrient, high color) and “mixotrophic” (high nutrient, high color) lakes.

Although metrics such as Trophic State Index^21^ gained popularity for providing instantaneous assessments of a lake’s autotrophic production, Williamson *et al*.^22^ encouraged a focus on NCP for lake classification given the importance of both nutrients and colored dissolved organic matter to lake structure and function. The NCP’s implementation is empirically supported by studies like Webster *et al*.^23^, where an analysis of ~1,600 temperate lakes in North America demonstrated that within lakes grouped by total phosphorus concentration (i.e., oligotrophic, mesotrophic, or eutrophic), those with ‘browner’ color (indicative of dissolved organic matter) had higher volumetric chlorophyll-a concentrations and shallower Secchi disk depths. A similar pattern was observed by Nürnberg and Shaw^43^, which analyzed 600 lakes spanning a latitude of 39**°**S to 82**°**N.

Here, we used the thresholds published in Webster *et al*.^23^ to classify lakes in the NLA dataset. Lakes were described as oligotrophic or ‘blue’ if total phosphorus concentration was less than 30 μg/L and true color was less than 20 platinum cobalt units (PCU), eutrophic or ‘green’ if total phosphorus was greater than 30 μg/L and true color was less than 20 PCU, dystrophic or ‘brown’ if total phosphorus was less than 30 μg/L and true color was greater than 20 PCU, and mixotrophic or ‘murky’ if total phosphorus was greater than 30 μg/L and true color was greater than 20 PCU (Fig. 1). Thresholds for total phosphorus are based on long established and widely accepted ranges affecting primary productivity^18^. True color thresholds are derived from Nürnberg and Shaw^43^. Eutrophic and mixotrophic classifications were combined into a single grouping due to similar spectral characteristics (see Step 3). Notably, the NCP assumes that phosphorus is the limiting factor for primary production. While there are instances where nitrogen can be the limiting nutrient^44,45^, ecosystems with low concentrations of total phosphorus also tend to have low total nitrogen concentrations^46^.

### Step 3: Create a training dataset

First, to create a dataset of lakes with *in situ* LTS measurements, we aggregated all total phosphorus and true color measurements from the U.S. EPA NLA 2007, 2012, and 2017 data (Figs. S1–3, Table S1). Although the NLA includes lakes smaller than 10 ha, we only used lakes of at least 10 ha in area for consistency with the HydroLAKES database. We then assessed the extent to which seasonal shifts in total phosphorus concentrations and true color values may alter interpretation of trophic state for a given lake using the subset of lakes that were sampled intra-annually. For lakes that were sampled multiple times within a U.S. EPA NLA campaign, we calculated the percentage of lakes that transitioned between trophic states within a single year and found that lakes broadly remained in the same NCP trophic state throughout a given summer (85.1% of lakes). Of the lakes that changed trophic state during a sampling season (14.9%), the majority transitioned from oligotrophic (61.5% of changing lakes; 8.7% of all lakes) or dystrophic (15.4% of changing lakes; 2.2% of all lakes) to eutrophic/mixotrophic. Few lakes transitioned from oligotrophic to dystrophic (15.4% of changing lakes; 2.2% of all lakes), and even fewer transitioned to oligotrophic from either dystrophic (3.9% of changing lakes; 0.5% of all lakes) or eutrophic/mixotrophic (3.9% of changing lakes; 0.5% of all lakes). No lakes transitioned from eutrophic/mixotrophic to dystrophic across all three NLA campaigns. Broadly, lakes transitioned between trophic states when lakes were located near a threshold for trophic state delineation (15–45 μg/L total phosphorus or 11–29 PCU). These results mirror those in Leech *et al*.^24^ and suggest that despite some lakes changing trophic states within a summer, the majority of lakes do not transition and those that do transition usually fall along an edge of a NCP-determined trophic state. Thus, for lakes sampled twice in one sampling campaign, we averaged total phosphorus and true color estimates.

Second, to match the *in situ* trophic states with remotely sensed imagery, we merged the complete 2007, 2012, and 2017 NLA dataset with the LimnoSat-US dataset^35^, where each NLA lake-year had corresponding Landsat spectral data. Because the NLA is designed to describe lakes’ summertime conditions, we filtered LimnoSat-US observances for those only occurring in June, July, and August, which we *a priori* defined as the summertime season for the contiguous U.S.; then, to create a characteristic reflectance for a given lake-year, we computed each lake-year’s median summertime reflectance for red, blue, green, and near-infrared bands. Because LimnoSat-US compiles reflectance values from Landsat 5, 7, and 8, there are differences in the number of images per lake and year. In particular, images from 1984 through 1998 were solely collected from Landsat 5, when lakes averaged 3.04 images per summer (minimum average images: 2.43 images; maximum average images: 3.64 images). From 1999 through 2012, summertime imagery was gathered from Landsat 5 and 7, when lakes averaged 5.64 images per summer (minimum average images: 3.37 images; maximum average images: 6.42 images). From 2013 through 2019, summertime imagery was collected from Landsat 7 and 8, when lakes averaged 5.42 images per summer (minimum average images: 4.87 images; maximum average images: 5.87 images).

Third, to better characterize spectral bands’ relative reflectance, we normalized each lake’s median summertime reflectance for the red, green, blue, and near-infrared band by the sum of the summertime reflectance values of all four bands. This normalization allowed us to differentiate lakes by trophic state based on their most prominent reflectances. For example, we anticipated that oligotrophic lakes would be dominated by high blue and green reflectances relative to the red and near-infrared bands. In contrast, dystrophic lakes would be dominated by the near-infrared band relative to green and red bands, because dystrophic lakes tend to have exceptionally low primary productivity and elevated dissolved organic matter. When assessing mixotrophic and eutrophic lakes, spectral characteristics were nearly identical, and to be conservative, we combined mixotrophic and eutrophic lakes into one category ‘eutrophic/mixotrophic’. These relative reflectances for all three lake trophic states were ultimately intended to discriminate among lakes that were optically similar in the visible spectrum (i.e., oligotrophic and dystrophic lakes). Notably, the decision to use median summertime relative reflectances differed from previous work^2^ that focused on the dominant wavelength, which is an aggregation of wavelengths detected in the visible spectrum and has been used to discriminate autotrophic production (i.e., blue vs green lakes), but not dystrophic states. Thus, our methods are better suited towards discriminating between oligotrophic and dystrophic lakes, because the dominant wavelength approach would consider both of these lake types to be “blue”.

### Step 4: Create classification models

To find an optimal performing classifier for lakes with unknown LTS, we employed three classification methods to predict trophic state: multinomial logistic regression^47^, extreme gradient boosting regression^48^, and a neural network using multilayer perceptrons^49^. Logistic regression is a parametric classification method, whereas gradient boosted regression and multilayer perceptrons are machine learning methods. The methods differ in how they make classifications. Using trophic state as a categorical response variable, logistic regression applies a linear regression of log-odds ratios to model the probability of a given trophic state for each lake. In contrast, gradient boosted regression applies decision trees to iteratively improve its predictions. Multilayer perceptrons apply a type of feedforward artificial neural network in which a backpropagation algorithm is used to subsequently update the individual weights of each neuron unit by comparing modeled predictions to the training data.

For each modeling method, we used z-scored, relative red, green, blue, and near-infrared reflectances as predictors. Model performance and potential for overfitting were assessed using a 90:10 train:test data split with spatial-holdout cross-validation. Initial hyperparameters for the gradient boosted regression and multilayer perceptron models were tuned by holding out 20% of each trophic class from the training observations to use for validation and conducting a coarse grid-search across the hyperparameter space. For each combination of hyperparameters, models were trained until validation performance did not increase for 20 consecutive epochs using categorical cross entropy as the objective function. During the multilayer perceptron hyperparameter tuning, we iterated through model fits using all combinations of 5, 10, and 20 hidden layers as well as a learning rate of 0.01, 0.001, and 0.0005. Multilayer perceptron hyperparameter tuning metrics were optimal for models with 20 hidden units and a learning rate of 0.001. During the gradient boosted regression hyperparameter tuning, we iterated through model fits using all combinations of 2, 3, and 4 maximum tree depths, subsample as well as column samples of 0.5 and 0.8, step sizes of 0.01 and 0.1, as well as a minimum child weight of 1 and 3. Gradient boosted regression hyperparameter tuning metrics were optimal for models with a max depth of 4, subsample of 0.5, column sample of 0.5, step size of 0.01, and minimum child weight of 1. For both multilayer perceptron and gradient boosted regression models, best performing hyperparameter tuning metrics were assessed by having lowest validation loss values.

These hyperparameters were then used in a spatial cross-validation routine^50^, where a given lake was held out as test data if it was included in the training data. During the spatial cross-validation routine, training data were divided into five folds, such that lakes within each test partition were not present in remaining training partitions (i.e., test metrics represent performance on unseen lakes). Training data within each fold were then partitioned into a 90:10 split with 10% of each trophic class set aside for an inner-loop fold validation. Models within each fold were trained using an early stopping criterion of 20 epochs to avoid overfitting on the training data. This inner-fold validation was additionally used to hypertune the best number of epochs for the final models. Finally, overall error metrics were calculated based on the mean prediction accuracy of the test partitions withheld from the inner-loop training of each fold. All reported metrics are based on the test partitions from the spatial cross-validation routine while final models were trained on the full dataset using the hyperparameters identified from the grid-search and inner-loop validation routines. We applied the final models to make predictions for all 55,662 lakes in the LimnoSat-US dataset.

### Step 5: Assess and compare model performance

To evaluate the final fitted models, we used test data predictions from the spatial-holdout routine to calculate each model’s overall and balanced accuracy, receiver-operator-characteristic (ROC) curves, as well as the area under the curve (AUC) of the ROC curve. Overall accuracy was calculated as the sum of true positives and true negatives divided by the total number of LTS predictions. Balanced accuracy was calculated as the sum of a true positive and true negative results for a single lake trophic state. Whereas overall accuracy can be biased towards more prevalent trophic states (i.e., eutrophic and oligotrophic lakes), balanced accuracy is useful to assess a model’s capacity to predict more rare trophic states (i.e., dystrophic lakes). As an additional metric of model performance, we calculated the AUC of each model’s ROC curve. The ROC curve visually graphs the relationship between the rate of a correct classification with the rate of a false classification. An AUC of 0.5 indicates a false prediction rate increases 1:1 with the rate of a correct prediction. AUCs greater than 0.5 imply a model performing better than random, even when a false positive rate is artificially inflated. Thus, comparing overall and balanced accuracy as well as ROC curves and AUCs allowed us to assess how models performed broadly as well as how robustly models predicted trophic state correctly.

Beyond model performance, we also evaluated whether model coefficients and variable importance for trophic state discrimination reflected NCP groupings. For increased interpretability across all three models, we employed SHAP (SHapley Additive exPlanation) analysis^51–53^ to better understand individual feature importance and influence in model predictions. SHAP analysis yields insight into the marginal contribution of a given feature (e.g., near-infrared spectra) on model output - in this case trophic state - and helps decode ‘black box’ results. Understanding the relative contribution of individual features in trophic state prediction not only helps explain feature roles in model accuracy and misclassification but also quantitatively connects features, such as remotely sensed data, to the biophysical parameters in which LTS prediction is grounded. SHAP feature contribution was calculated for blue, green, red, and near-infrared Landsat spectra. SHAP feature contribution was scored for oligotrophic, dystrophic, and eutrophic/mixotrophic classifications and across each of the three models. This scoring illuminates the relationship among feature values and SHAP contribution for a given trophic state classification and for a given model. Specifically, for classification problems, a positive SHAP value indicates that a given input contributed to a positive classification and a negative value indicates the input contributed to a low probability for a given classification.

## Data Records

The LTS-US dataset^54^ is available at the Environmental Data Initiative (10.6073/pasta/212a3172ac36e8dc6e1862f9c2522fa4) and is structured in a tabular format, where each row is a lake-year combination. The main dataset is contained in “ensemble_predictions.csv” and is structured in a way that provides both categorical LTS predictions as well as probabilities for each LTS prediction. The probabilities reported in “ensemble_predictions.csv” are averaged probabilities generated from each of the three modeling methodologies. An additional tabular dataset (“individual_predictions.csv”) contains probabilities generated for each of the three modeling methodologies and can be merged with “*ensemble_predictions*.csv” by the “Hylak_id” and “year” columns.

We provide raw and average predicted LTS probabilities as well as variance among models for a given LTS prediction to allow future users to filter predictions of a certain threshold for their particular analysis. Although many thresholds may exist, reporting probability thresholds used in subsequent analyses will help maintain reproducibility and synthesis across studies. Below, we detail column names and metadata for each of the core datasets contained within the LTS-US data product.

*“ensemble_predictions.csv”*

*Hylak_id*

HydroLAKES unique identifier of lake. Preserved from HydroLAKES input data to enable future merging with HydroLAKES attributes.

*year*

Year, spans 1984 through 2020.

*categorical_ts*

Categorical predicted lake trophic state (i.e., oligo, eu/mixo, dys). Categorical prediction is based on the highest probability among *mean_prob_dys*, *mean_prob_eumixo*, and *mean_prob_oligo*.

*mean_prob_dys*

Probability that a lake-year combination is dystrophic. Probability is calculated by averaging probabilities from all three modeling methods.

*mean_prob_eumixo*

Probability that a lake-year combination is eutrophic or mixotrophic. Probability is calculated by averaging probabilities from all three modeling methods.

*mean_prob_oligo*

Probability that a lake-year combination is oligotrophic. Probability is calculated by averaging probabilities from all three modeling methods.

*var_prob_dys*

Variance in probabilities among all three modeling methods that a given lake-year is dystrophic.

*var_prob_eumixo*

Variance in probabilities among all three modeling methods that a given lake-year is eutrophic/mixotrophic.

*var_prob_oligo*

Variance in probabilities among all three modeling methods that a given lake-year is oligotrophic.

*“individual_predictions.csv”*

*Hylak_id*

HydroLAKES unique identifier of lake. Preserved from HydroLAKES input data to enable future merge with HydroLAKES attributes.

*year*

Year, spans 1984 through 2020.

*prob_dys_mlr*

Probability that a lake-year combination is dystrophic. Probability is calculated by multinomial, multiple logistic regression.

*prob_eumixo_mlr*

Probability that a lake-year combination is eutrophic or mixotrophic. Probability is calculated by multinomial, multiple logistic regression.

*prob_oligo_mlr*

Probability that a lake-year combination is oligotrophic. Probability is calculated by multinomial, multiple logistic regression.

*prob_dys_mlp*

Probability that a lake-year combination is dystrophic. Probability is calculated by multilayer perceptron.

*prob_eumixo_mlp*

Probability that a lake-year combination is eutrophic or mixotrophic. Probability is calculated by multilayer perceptron.

*prob_oligo_mlp*

Probability that a lake-year combination is oligotrophic. Probability is calculated by multilayer perceptron.

*prob_dys_xgb*

Probability that a lake-year combination is dystrophic. Probability is calculated by a gradient-boosted regression.

*prob_eumixo_xgb*

Probability that a lake-year combination is eutrophic or mixotrophic. Probability is calculated by a gradient-boosted regression.

*prob_oligo_xgb*

Probability that a lake-year combination is oligotrophic. Probability is calculated by a gradient-boosted regression.

## Technical Validation

### Model performance diagnostics

To assess how each model correctly classified training data, we compared the model accuracies, balanced accuracies, and AUC of ROC curves. Overall and balanced model accuracies were similar, where all models had accuracies ranging from 72.4 to 72.9% and balanced accuracies ranging from 69.9 to 71.5%. AUCs of ROCs were likewise similar across all three model techniques, ranging from 0.88 to 0.90 (Figure S4). These combined metrics suggest that all three modeling approaches performed similarly, when assessing model performance with global metrics.

**Fig. 3:**
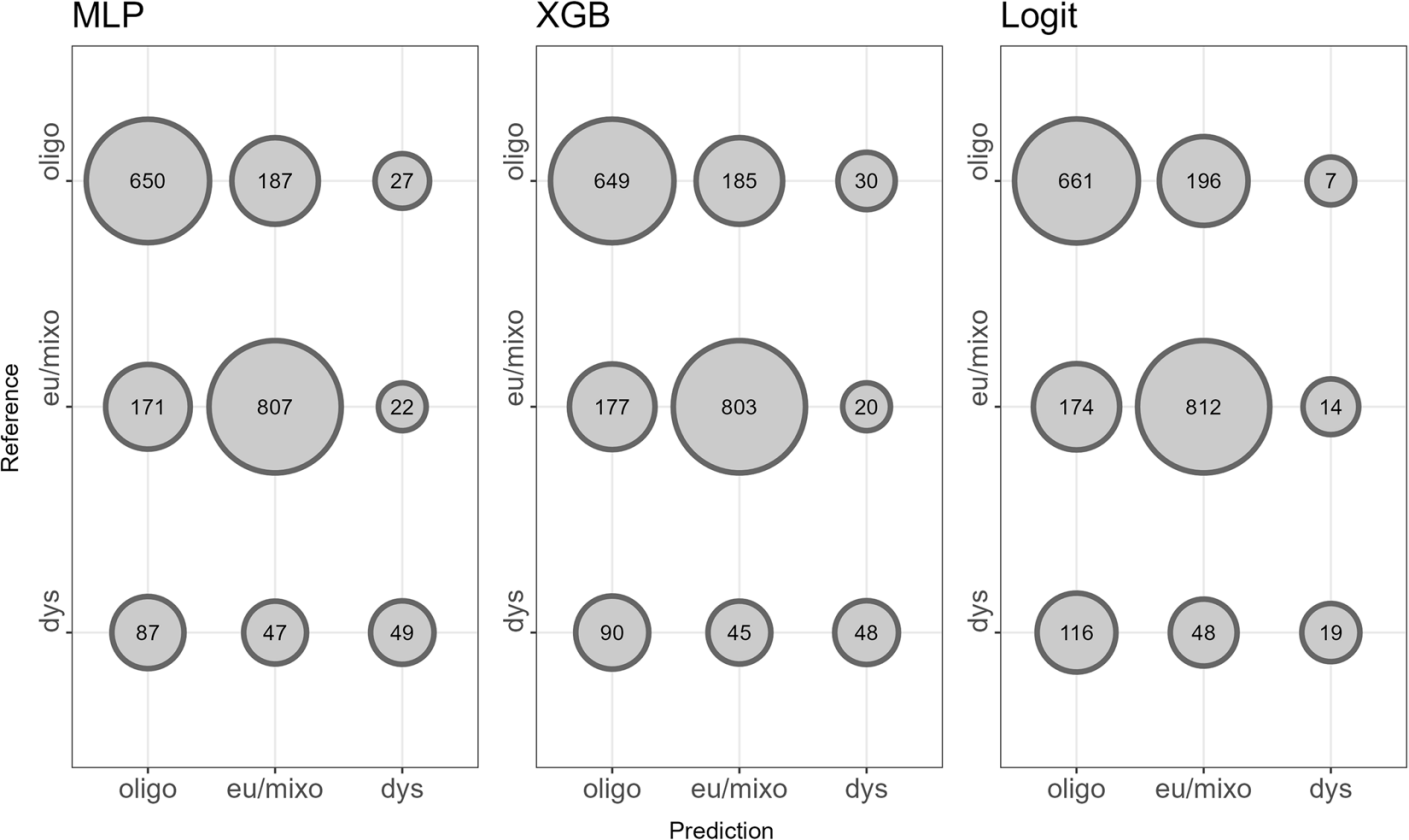
Confusion matrices from each modeling approach. Confusion matrices were generated using the test partitions for each spatial-holdout cross-validation. Circle size is scaled by the number of lakes falling within each category. Trophic states for model predictions and reference data correspond to the acronyms “dys” for “dystrophic”, “eu/mixo” for “eutrophic/mixotrophic”, and “oligo” for “oligotrophic”. Model acronyms are located as the title for each confusion matrix, where “MLP” signifies “Multilayer Perceptron”, “XGB” signifies “Gradient Boosted Regression”, and “Logit” signifies “Multinomial Logistic Regression”.

Although models performed similarly at high levels, they varied more in their robustness to classify dystrophic lakes (Fig. 3). Machine learning-based methods, such as multilayer perceptron (60%) and gradient boosted regression (58%), had higher balanced accuracies, whereas distribution-based methods, such as logistic regression (55%), had lower balanced accuracies. These differences were largely driven by deviations in true positive rates (47.5–50.6%), whereas true negative rates were higher (91.8–92.7%). This difference in true negative and true positive rates is likely due to spectral similarities between oligotrophic and dystrophic lakes, where both are characterized by low primary production in comparison to eutrophic/mixotrophic lakes. Although these differences only span 5%, they may be important, given that dystrophic lakes tend to be uncommon relative to oligotrophic and eutrophic lakes^23^. Such differences imply variation in each model’s robustness to predict rarer trophic states, but our overall metrics of model performance highlight exceptional congruence across all three modeling techniques.

### Spatial patterns in lake classification

To evaluate spatio-temporal patterns in trophic state classification, we created spatial confusion matrices, where predictions and reference sites were plotted across the entire United States. We *a priori* hypothesized that when misclassifications result from lake-specific deviations, misclassifications should be distributed throughout the United States without any clear spatial patterns. In the event that spatial clustering of misclassified lakes occurred, these patterns should be more pronounced where high densities of a given lake trophic state are located. In cases when lake clustering appears in an unexpected area, these patterns should be more attributed to place-based irregularities in spectral data.

Confusion between oligotrophic and eutrophic/mixotrophic lakes were spatially distributed throughout the entire continental United States, with no evidence of spatial clustering (Figures S5–S7). In contrast, dystrophic misclassifications were broadly isolated to the Upper Midwest and Upper Northeast regions. Consistent with our hypotheses, these regions are associated with increased densities of dystrophic lakes, suggesting that optical similarities between oligotrophic and dystrophic lakes in these regions may lead to increased misclassification. Notably, dystrophic lakes tended to be misclassified as oligotrophic, whereas oligotrophic lakes tended to not be misclassified as dystrophic, meaning that our predictions should be conservative with assigning an individual lake as dystrophic.

### Assessing patterns in lake classification

Given that lake trophic state classifications may be a product of a lake’s limnological, morphological, and geographic properties, we performed a series of analyses of variance (ANOVA) to test for significant differences (i.e., p-value < 0.05) in lake classification accuracy. For each ANOVA, a lake property was the response variable, and predictors were lake trophic state, model correctness (i.e., correct or incorrect classification), and model type. All response variables were log-transformed to approximate a normal distribution. Because each analysis had an unbalanced sample size, we calculated Type II Sum-of-Squares^55^. Residuals for each model were assessed for normality and homogeneity of variance.

The main goal of each ANOVA was to assess whether variation in a lake parameter could be associated with variation in model methodologies, model correctness, or trophic states themselves. Consequently, our ANOVAs do not include interaction terms, as most interactions would not be helpful for understanding patterns in how our classification models performed.

### NCP patterns in lake classification

**Fig. 4:**
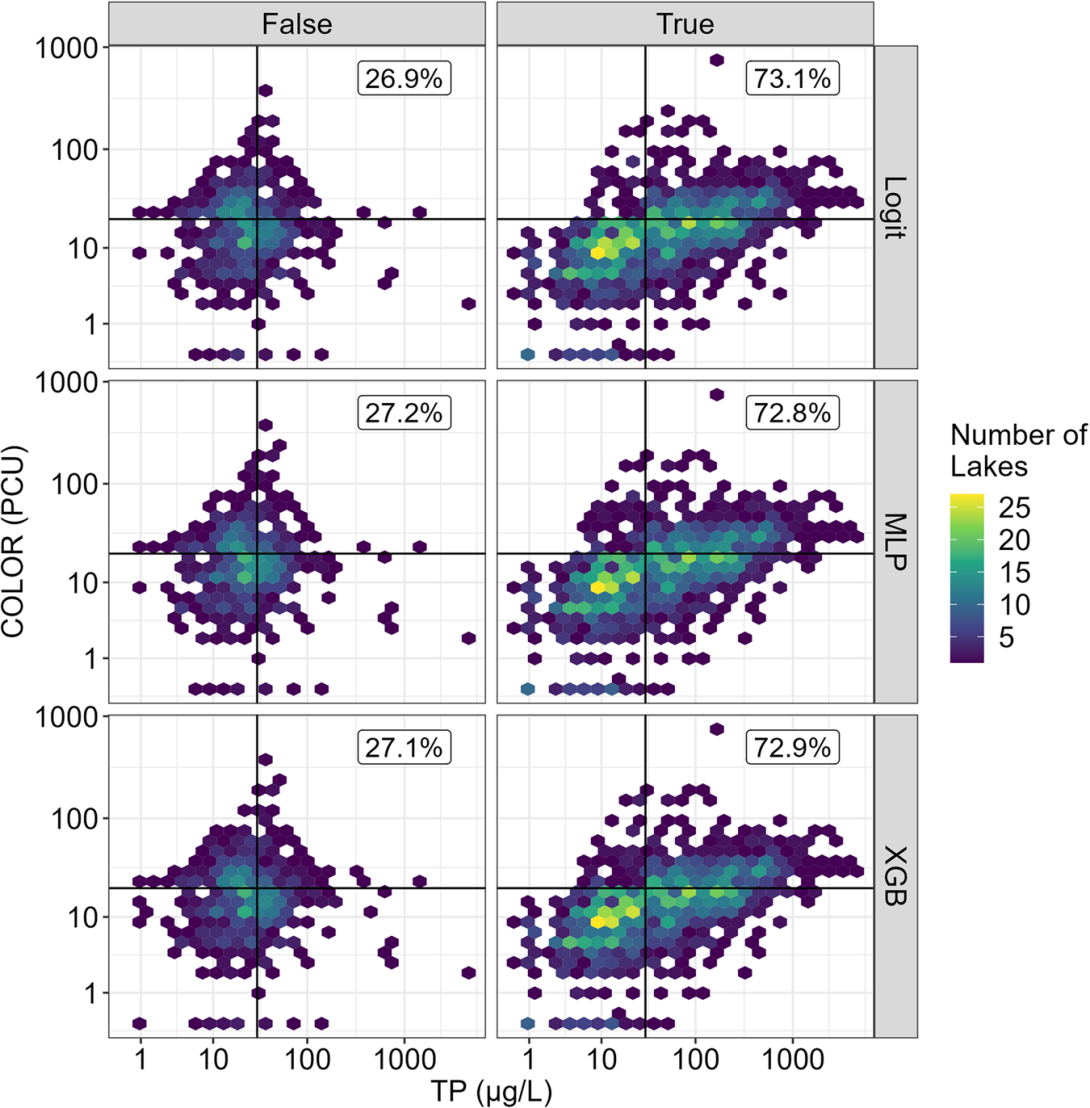
True and false classifications from testing data displayed on the NCP axes. Hexbins are colored by the number of lakes they contain. Labels reflect the percentage of lakes correctly or incorrectly predicted within a given modeling technique. Incorrect LTS predictions tend to be located at the nexus of trophic state groupings. Correct predictions tend to more accurately reflect the breadth of ranges that can be observed within each of the LTS groupings.

**Fig. 5:**
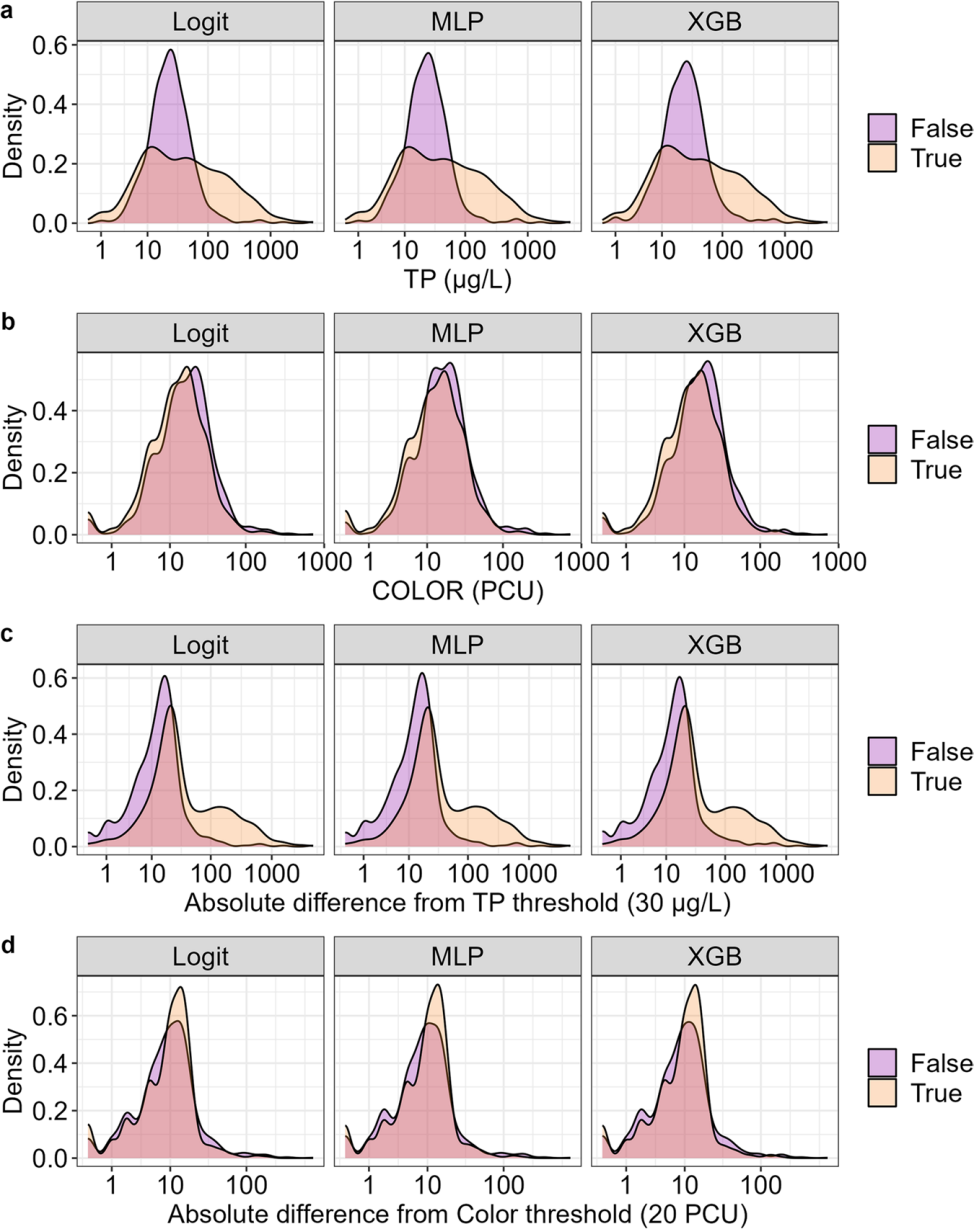
Density plots for total phosphorus (**a**) and true color (**b**) values as well as absolute differences from NCP thresholds for total phosphorus (**c**) and true color (**d**) among correctly (i.e., True; orange) and incorrectly (i.e., False; purple) classified trophic states. Because misclassifications appeared to increase in frequency near threshold values for trophic state classification, we also assessed classification accuracies across absolute differences for each variable and threshold value. Across all models, we noticed that misclassifications tended to be highest near NCP thresholds for total phosphorus and color. Total phosphorus concentrations of 15–45 μg/L tended to be associated with false classifications. True color concentrations of 11–29 PCU tended to be associated with a false classification.

To assess how a lake’s misclassification may be related to its position in the NCP, we assessed where correctly and incorrectly classified lakes were located in the NCP. Lakes that were incorrectly classified tended to be located near total phosphorus (30 ± 15 μg/L) and color (20 ± 9 PCU) thresholds, with a large portion at the nexus of the total phosphorus and color thresholds (Fig. 4). Across all modeling techniques, correctly classified lakes spanned a wider range across both axes, especially total phosphorus. Median total phosphorus concentration for misclassified lakes was 24 μg/L (range: 1–4,772 μg/L), whereas median total phosphorus concentration for correctly classified lakes was 36 μg/L (range: 0.24–4,144 μg/L). Similarly, median PCU for correctly (14 PCU; range: 0–724 PCU) and incorrectly (16 PCU; range: 0–350 PCU) classified lakes were along the edge of the color threshold of 20 PCU. When assessing total phosphorus and color independently, ANOVA suggested that total phosphorus concentrations were significantly different for correctly and incorrectly classified lakes, whereas differences in color were not significantly different across correctly and incorrectly classified lakes (Table 1; Fig. 5).

**Table 1:** ANOVA table for total phosphorus and true color measurements in response to model type, model correctness, and trophic state.

	Sum-of-Squares	Degrees of Freedom	F-value	P-value
**(A) Total Phosphorus**				
Model	0	2	0.003	0.997
Correct	5.62	1	43.64	<0.001
Trophic State	1,335.42	2	5,183.4	<0.001
**(B) Color**				
Model	0	2	0	1
Correct	0	1	0.02	0.89
Trophic State	286.62	2	1521.80	<0.001

ANOVAs were assessed with Type II Sum-of-Squares to account for unbalanced sample sizes. To approximate a normal distribution, both total phosphorus and color were log-transformed. A p-value threshold of 0.05 was used to assess significance for each predictor.

Beyond total phosphorus and color patterns influencing lake classification, our analyses of lakes that transitioned trophic states within a summer suggest that lakes along a NCP boundary (i.e., near total phosphorus or color threshold) are more prone to misclassification. Among lakes that transitioned within a summer, the most frequent change in lake trophic state was among lakes switching from oligotrophic to eutrophic (61.5% of NLA lakes that changed in a summer; 8.7% of all NLA lakes). Considering that both total phosphorus concentrations as well as summertime lake phenologies are associated with algal production and can cause a lake to transition categories within a summer, our results of NCP patterns are not surprising. Rather, confusion along the total phosphorus axis of the NCP, an axis that corresponds with autotrophic productivity, is concordant with the idea that a lake can experience moments of eutrophy - e.g., a pulse of nutrients or algal growth - while otherwise being oligotrophic for the majority of the summer. Therefore, classifications made for lakes at the boundary of trophic states can be challenging, and our validation analyses describe total phosphorus and color conditions where misclassifications may be more common.

### Morphological and locational patterns in lake classification

At the spatial resolution of Landsat’s sensors, there is a risk of “mixed pixels”, where a pixel includes water with fractions of adjacent bare land or vegetation. Given the difference in optical contrast between water and other features, even minor differences can lead to large errors in estimating surface reflectance. A major source of uncertainty in lake optical water quality estimation is the separation of water and atmospheric effects^56^. The latter increases in severity near land, and this adjacency effect can extend several kilometers, depending on the state of the atmosphere.

Before assessing how edge and lakebed effects may influence model classifications, we first ensured that spectral differences between each trophic state in our dataset were greater than differences within a trophic state when accounting for lake area, depth, and shape. To evaluate how edge and lakebed effects may be present within our training and test data, we used lake area, average depth, and shoreline development (a metric of how closely a lake’s shape resembles a circle) data from HydroLAKES^33^ as well as maximum depth from the GLOBathy dataset^57^. While evaluating lake area, we noticed that smaller lakes tended to have higher near-infrared relative reflectance values, and relative near-infrared reflectance generally decreased with increasing lake area (Figures S8–S10). Because LimnoSat-US aggregates reflectance data at the lake’s Chebyshev center, the point in the lake farthest away from shore, smaller lakes would likely have Chebyshev centers that are closer to the shoreline. As terrestrial near-infrared reflectances tend to be higher than aquatic near-infrared reflectances, smaller lakes with Chebyshev centers closer to the shoreline may be associated with increased near-infrared signatures. Similarly, relative blue reflectance increased with increasing lake surface area, which would likewise be expected, as larger lakes likely have a Chebyshev center that is farther from shore, and therefore, less influenced by shoreline effects. With respect to lakebed effects, the shallowest lakes tended to have slightly elevated relative green reflectance, which would be consistent with increased primary production. Across all trophic states, lakes with average depths of 1–10 m were also associated with increased relative near-infrared reflectance, suggesting that these lakes may have the highest near-infrared reflectance due to reflectance signatures of lakebed substrate or increased benthic algal production.

To evaluate how models might misclassify lakes in response to morphological, geographic, and biological characteristics, we examined how lake depth, elevation, surface area, shoreline development, and mean chlorophyll concentration may correspond to correct and incorrect classifications. Average and maximum lake depth can be used to evaluate a lake’s potential for lakebed effects, where reflectance from benthic algae, emergent vegetation, or sediment may confound signals for the actual surface of the lake. Assessing classification differences across elevation ranges can be important for understanding atmospheric effects on reflectance data, where higher elevations may have fewer aerosols, and therefore contain fewer misclassifications. Examining misclassifications across lake sizes can reveal potential for adjacency effects, where surrounding geologies or vegetation may obscure surface reflectances observed over the lake. Shoreline development can likewise reveal adjacency effects, where lakes with more complex shapes but with large areas may be prone to misclassification. Lastly, chlorophyll a concentrations can inform that our models are capturing patterns expected through how we operationally defined LTS, where higher chlorophyll concentrations should be observed in eutrophic/mixotrophic lakes relative to dystrophic and oligotrophic lakes.

**Fig. 6:**
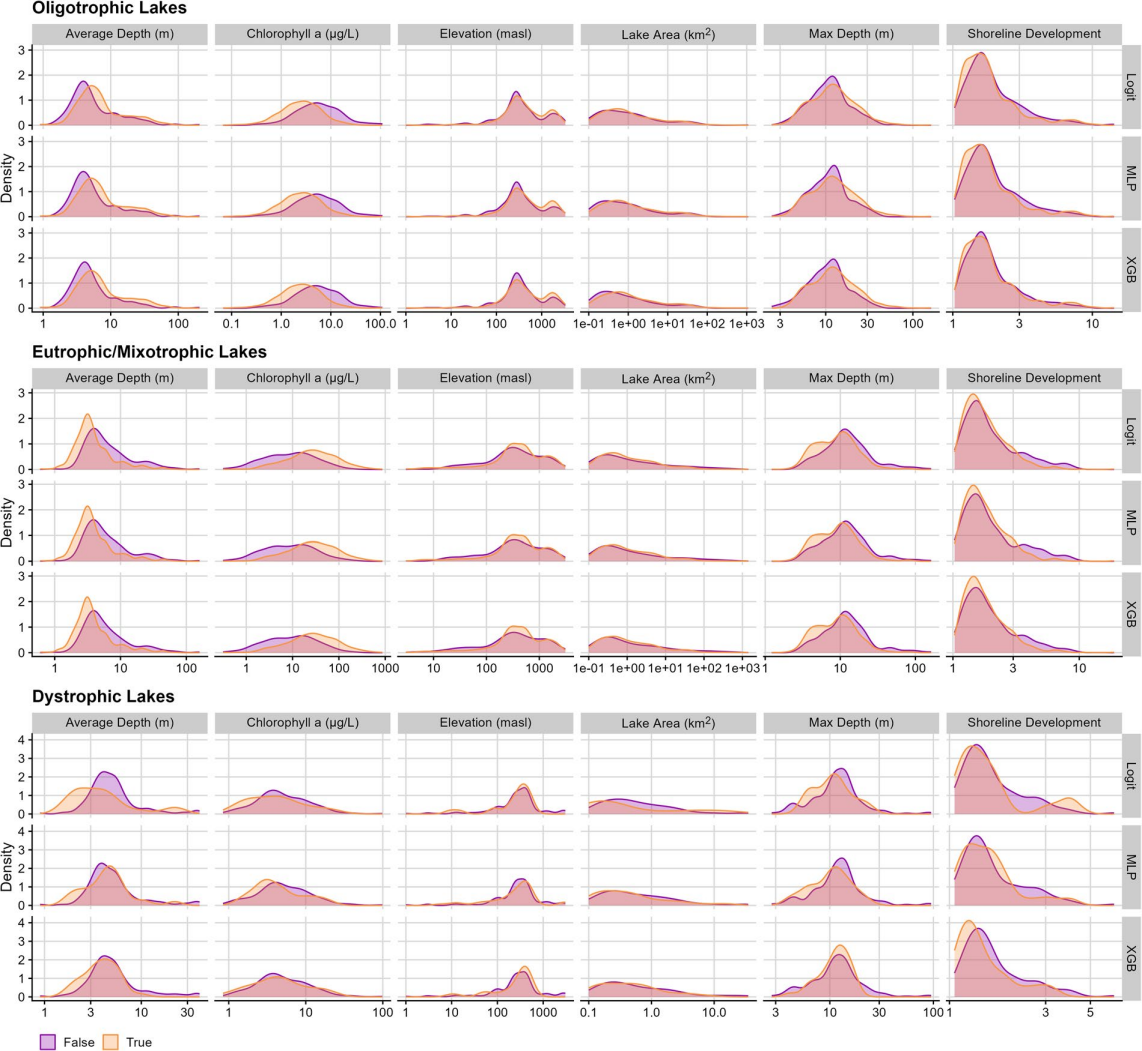
Density distributions for each lake’s average depth, mean summertime chlorophyll a concentration, elevation, area, maximum depth, and shoreline development values across true (orange) and false (purple) classifications. Values are log-transformed to show characteristic density distributions over a wide range in value magnitudes. In general, patterns across true and false classifications were consistent across all three types of models. Depth was a primary characteristic for misclassified oligotrophic and eutrophic/mixotrophic lakes, where shallower oligotrophic and deeper eutrophic/mixotrophic lakes tended to be misclassified.

ANOVA results suggested that average depth, chlorophyll a, maximum depth, shoreline development, and elevation differed significantly across correct and incorrect misclassifications (Table 2), although differences based on average and maximum depth as well as chlorophyll a were more visually apparent than those observed for elevation and shoreline development (Fig. 6). In contrast, lake area did not differ significantly across correct and incorrect classifications (Table 2).

**Table 2:** ANOVA table for lake morphological and locational properties in response to model type, model correctness, and trophic state.

	Sum-of-Squares	Degrees of Freedom	F-value	P-value
**(A) Lake area**				
Model	0	2	<0.001	1
Correct	0.4	1	0.76	0.36
Trophic State	35.2	2	33.17	<0.001
**(B) Average Depth**				
Model	0	2	0.001	1
Correct	1.61	1	14.94	<0.001
Trophic State	54.32	2	251.53	<0.001
**(C) Maximum Depth**				
Model	0	2	0.001	1
Correct	1.2	1	16.86	<0.001
Trophic State	14.17	2	99.53	<0.001
**(D) Elevation**				
Model	0	2	0.001	1
Correct	3.06	1	10.61	0.001
Trophic State	15.39	2	26.63	<0.001
**(E) Shoreline Development**				
Model	0.0	2	0.002	1.00
Correct	0.726	1	22.33	<0.001
Trophic State	2.09	2	32.16	<0.001
**(F) Mean Chlorophyll Concentration**				
Model	0.0	2	0.000	1.00
Correct	2.88	1	11.40	<0.001
Trophic State	1010.73	2	1998.29	<0.001

ANOVAs were assessed with Type II Sum-of-Squares to account for unbalanced sample sizes. To approximate a normal distribution, all response variables were log-transformed. A p-value threshold of 0.05 was used to assess significance for each predictor.

Together, these analyses suggest that lakebed reflectance may lead to lake trophic state misclassification, whereas edge effects are likely less consequential for inaccurate lake trophic state classifications. In particular, shallower oligotrophic lakes (i.e., average depth <5 m and maximum depth <15 m; Fig. 6) and deeper eutrophic lakes (i.e., average depth >5 m and maximum depth >15 m; Fig. 6) tended to be misclassified. We speculate that these differences may stem from shallower, oligotrophic lakes having pronounced benthic algal growth^58^ or emergent macrophytes that can produce a strong green signal. Conversely, deeper eutrophic lakes may have less concentrated algal growth in the water column, thereby creating a stronger blue reflectance relative to green reflectance and increasing chances for misclassification (see *Optical patterns in lake classification*). These differences may also be related to chlorophyll a concentration, where oligotrophic lakes with higher concentrations tended to be classified as eutrophic/mixotrophic, and eutrophic/mixotrophic lakes with lower concentrations tended to be misclassified as oligotrophic (Fig. 6). Overall, these results correspond with our NCP validation analyses, where total phosphorus concentrations were associated with greater misclassifications of oligotrophic lakes as eutrophic. Given the potential for lakes to be misclassified because of issues with lakebed reflectance, considering whether depth could alter results and building analytical workflows to assess sensitivity to interference from lakebed reflectance (see *SHAP Analysis* for more detail) could improve model lake classifications.

### Optical patterns in lake classification

To evaluate how models might misclassify lakes based on reflectance values, we assessed how z-scored relative red, green, blue, and near-infrared reflectance values differed between correctly and incorrectly predicted lake trophic state. Because we used relative reflectances that are inherently interdependent, and thus violate ANOVA assumptions, we elected to forgo significance tests for whether band ranges differed across modeling methods.

**Fig. 7:**
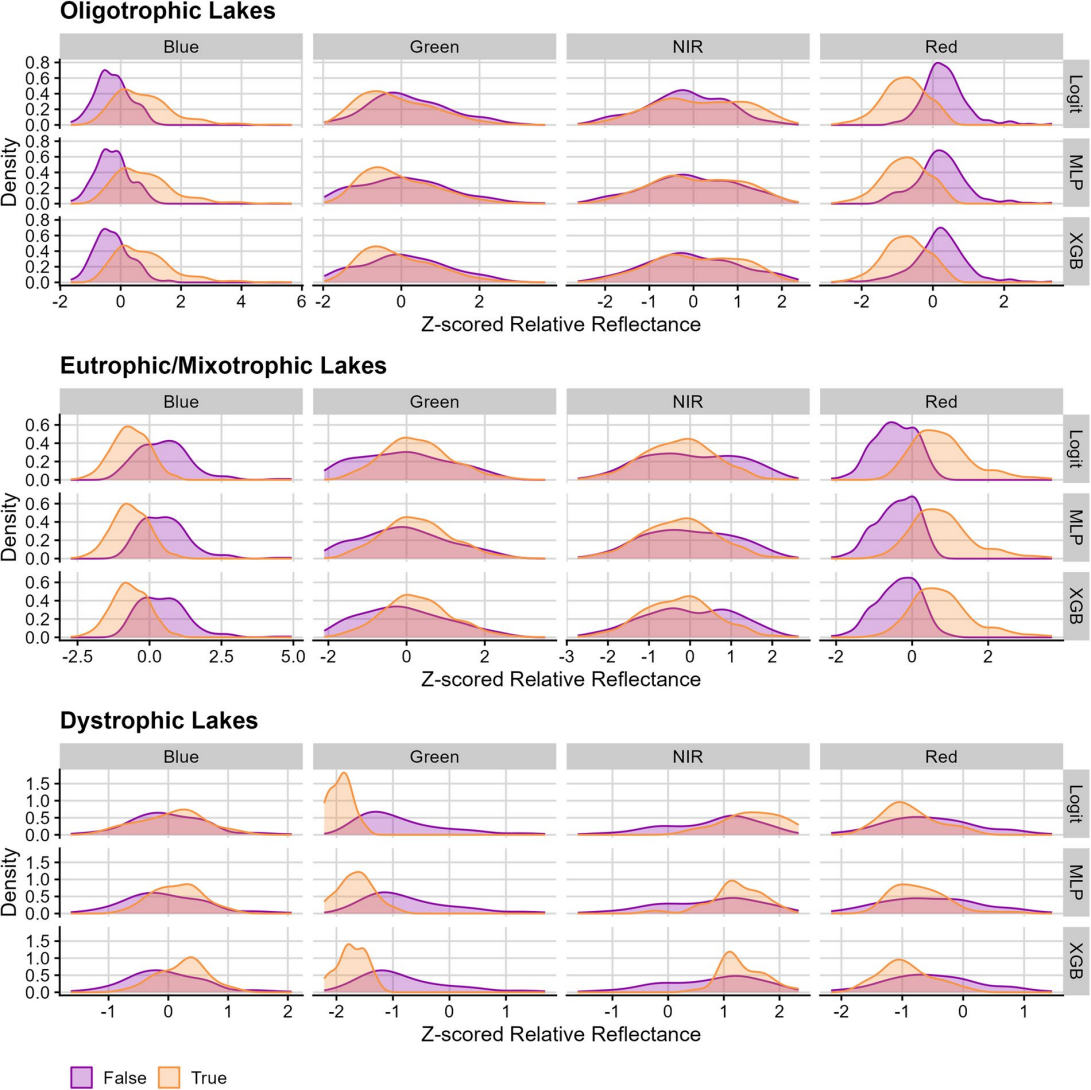
Density distributions for each Landsat band’s z-scored, relative reflectance value across true (orange) and false (purple) classifications. In general, patterns across true and false classifications were consistent across all three types of models. Oligotrophic lakes tended to be misclassified when red bands were high and blue bands were low. Conversely, eutrophic/mixotrophic lakes tended to be misclassified when blue bands were high, and red bands were low. Dystrophic lakes tended to be misclassified when near infra-red bands were low and when green bands were high.

For dystrophic lakes, incorrectly classified lakes, compared to correctly classified lakes, tended to have lower z-scored near-infrared and blue band values as well as higher green and red values (Fig. 7). For eutrophic/mixotrophic lakes, misclassified lakes tended to have lower values for red and green bands as well as higher values for blue bands (Fig. 7). For oligotrophic lakes, incorrectly classified lakes tended to have higher red and lower blue band values (Fig. 7).

These inconsistencies in LTS classification correspond with variation that can be present in natural systems. Dystrophic lakes are generally characterized as having low primary productivity and high dissolved organic matter, which should result in low green band values as well as higher near-infrared values, yet misclassified dystrophic lakes tended to have low near-infrared as well as high red and green bands. Eutrophic and mixotrophic lakes are generally characterized as having high productivity, which should result in high green values, yet misclassified eutrophic and mixotrophic lakes tended to have low green and red as well as high blue bands. Oligotrophic lakes should be characterized as having high blue bands, yet misclassified lakes tended to have low blue and high red bands, which may be a product of bottom reflectance. Together, these misclassifications likely represent lakes that are not characteristic of LTS classifications. For example, a more productive oligotrophic lake could produce a stronger red and green signature and, therefore, be classified as eutrophic. Likewise, less productive eutrophic lakes may be optically more similar to oligotrophic lakes and, therefore, be characterized by lower red and green bands.

### SHAP Analysis

To evaluate the influence of remote sensing reflectance inputs on final predictions, we assessed the distribution of SHAP values calculated for each predictor and for each trophic state. In general, SHAP values can be useful for decoding how machine learning and parametric methods may assign relative importance to a given predictor, thereby increasing interpretability of a model. In an instance where models are classifying lakes based on *a priori* hypothesized relationships, SHAP values across predictors should correspond to the *a priori* hypothesized relationships. For example, oligotrophic lakes are generally characterized as having high blue reflectance relative to red and green, and in a case where models reflect this understanding, SHAP values should attribute an oligotrophic classification to high values in the relative blue reflectance. Consistently high attributions for blue reflectances should subsequently result in high overall feature importance when discriminating oligotrophic lakes.

When evaluating feature importance across trophic states, measured as the mean absolute SHAP value of a given feature, all models agreed on the most influential features for classification (Figure S11). Furthermore, the distribution of SHAP values reflected limnological understanding of each trophic state’s inherent properties. For dystrophic lakes, SHAP values indicate that models relied on low green and high near-infrared and red band values, corroborating the idea that dystrophic lakes should have lower primary production and increased cDOM^22,59^ (Figure S11). Predictions for eutrophic and mixotrophic lakes were attributed to high red and low blue band values, corresponding with the idea that eutrophic and mixotrophic lakes should have higher algal production^24^ (Figure S11). Conversely, SHAP values for oligotrophic lakes attributed predictions to low red and high blue band values, agreeing with the idea that oligotrophic lakes should have lower algal production^24^ (Figure S11). Beyond each individual trophic state’s most important predictors, our SHAP analysis mirrored the logic of NCP analyses, where lakes with lower true color values (i.e., oligotrophic and eutrophic) were discriminated more effectively by bands associated with autotrophic capacity, whereas lakes with higher true color values (i.e., dystrophic) were discriminated more effectively by bands suggesting decreased autotrophic production and increased colored dissolved organic matter.

SHAP values can also provide insight on what drives models to misclassify certain lakes. Specifically, when examining smaller, shallower oligotrophic lakes that could potentially be influenced by bottom reflectance or adjacency effects, we observed that some misclassifications were attributable to models relying on low relative blue reflectance and high relative near-infrared reflectance (Figures S12–S23). These patterns indicate that in certain lakes, the models were unable to distinguish the spectral signatures that are potentially attributable to sediment or benthic algae as well as shoreline vegetation and soil. The spectral similarity between shallow oligotrophic and deep eutrophic lakes is relevant to active research trajectories in limnology, particularly those examining the relatively high contributions of benthic algal communities to whole lake productivity in oligotrophic lakes^58,60–64^. Given the potential for lakebed effects to alter classifications, research questions could consider the influence of depth-related misclassifications.

### Comparing predicted and NLA spatial patterns

To independently validate the LTS-US dataset’s robustness in capturing macroscale and multi-year changes in lake trophic state, we replicated analyses similar to Leech *et al*.^24^ and compared statistics from the NLA with those from the LTS-US dataset. We first merged the lake trophic state classifications from the 2007, 2012, and 2017 NLA campaigns as well as the LTS-US dataset with the U.S. EPA Level I Ecoregions^30^. We then calculated the proportion of each trophic state occurring within each ecoregion in a given year. To compare the NLA and the LTS-US dataset, we calculated the absolute difference between predicted and estimated proportions for each trophic state within each year and ecoregion.

**Fig. 8:**
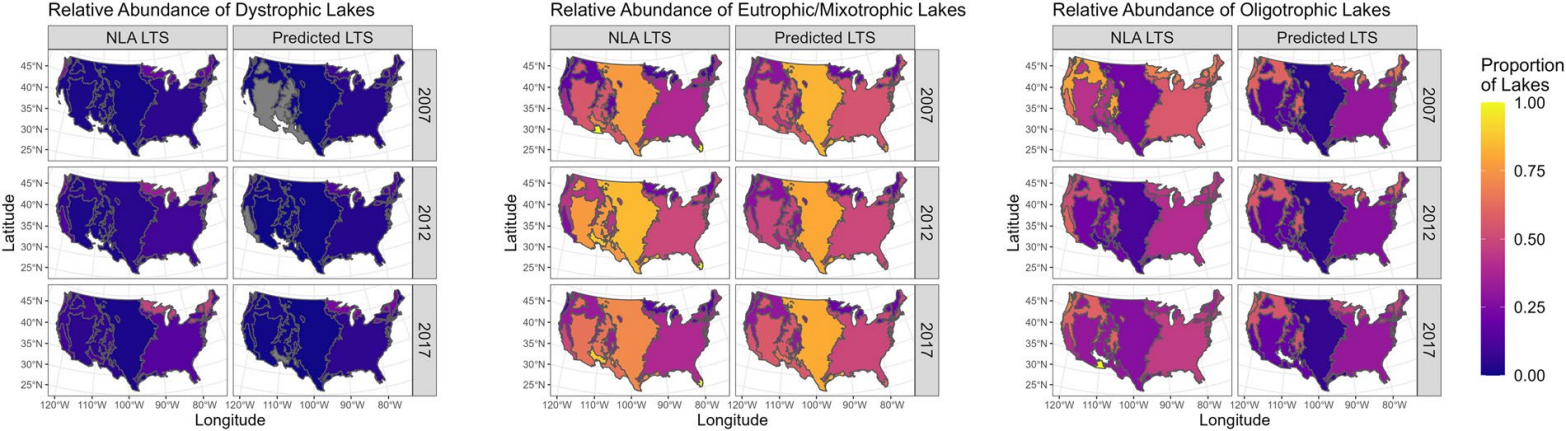
National-scale maps of U.S. Environmental Protection Agency Level I Ecoregions colored by proportion of lakes occurring in that ecoregion. For each trophic state, we compare estimated trophic state relative abundance from the NLA with predicted proportions from the ensemble LTS-US dataset.

Predicted and measured proportions were broadly consistent across all three years. Visually, all three years and trophic states followed consistent trends across all ecoregions (Fig. 8). For example, our models generally captured increasing dystrophic and decreasing oligotrophic lakes in northern forested regions, a pattern consistent with Leech *et al*.^24^. Absolute differences between estimated and predicted proportions across ecoregions were likewise congruent across all three years. Eutrophic/mixotrophic lakes tended to have the smallest differences (mean = −5.3%, sd = 19%), indicating that our models may overestimate relative abundance of eutrophic and mixotrophic lakes (Figure S24). In contrast, dystrophic (mean = 7.0%, sd = 6.7%) and oligotrophic (mean = 7.6%, sd = 22.4%) relative abundance tended to be underestimated (Figure S24). Larger standard deviation values were caused by some ecoregions having few lakes overall, thereby increasing proportions of a given trophic state within an ecoregion. When filtering for ecoregions that contained at least 10 lakes, we noticed similar patterns of eutrophic and mixotrophic lakes being slightly overestimated (mean = −7.9%, sd = 11.3%), as well as dystrophic (mean = 9.1%, sd = 6.7%) and oligotrophic (mean = 4.6%, sd = 13.8%) lakes being underestimated; yet the standard deviation in absolute differences decreased.

Together, these analyses demonstrate that though the LTS-US dataset does contain biases towards eutrophic/mixotrophic classification, its overall congruence with the NLA highlights its robustness. These biases may stem from our models attempting to classify lake ecosystem properties based on optically visible (i.e., color) and optically invisible (i.e., phosphorus) properties, where the exceptionally oligotrophic, dystrophic, and eutrophic/mixotrophic lakes are more consistently discriminated. In contrast, the NLA may likewise contain biases due to site selection, whereas our methods select for all lakes of at least 10 ha in area. Regardless of the biases in the LTS-US and NLA datasets, the congruence between the two is even more notable considering that our modeling approaches and the NLA use independent methods for classifying lake trophic state. The NLA uses *in situ* total phosphorus and true color measurements, whereas our methods use lake red, green, blue, and near-infrared reflectance. Furthermore, despite not including temporal or spatial predictors, our models reproduce NLA spatial and temporal trends in lake trophic state at larger spatial and temporal scales.

Given both the potential biases and robustness of the LTS-US data product, cross-referencing the LTS-US dataset with known trends in an area of interest, especially in areas where lakes may be less abundant, could enhance regional and local analyses. In instances where the LTS-US dataset may be more biased, reproducing the LTS-US dataset using both our existing code and particular predictors of interest for a region, such as average depth, lake area, or watershed area could offer particular insights into why a given region may be more prone to misclassifications. Creating tailored versions of the core LTS-US dataset can promote further understanding of features that may be important for assessing lake trophic state with remotely sensed surface reflectance data.

### Manual quality control

To ensure integrity of lake classifications across all steps of our pipeline, we randomly subsampled 250 lakes from the final dataset and manually cross-referenced their predicted trophic state with independent sources. The random subsample only included lakes that had associated names in the HydroLAKES dataset and was stratified by lake surface area, where surface areas were binned by orders of magnitude (i.e., <1 km^2^, (1, 10] km^2^, (10, 100] km^2^, (100, 1,000] km^2^, (1,000, 10,000] km^2^, >10,000 km^2^). We filtered specifically for lakes with names because we assumed that named lakes within the HydroLAKES database would likely have more publicly available information about their water quality and would likely be easier to find within managerial reports and scientific publications.

To minimize bias, persons engaged in manual checking only received lake latitude and longitude, name, and the U.S. state where the lake was located. All persons engaged in manual checking were not involved in model and prediction development and were, therefore, blind to individual lake predictions. When possible, persons identified trophic states for multiple years, although many sources only referenced a lake’s trophic state in an individual year or broadly across multiple years. In either case, LTS was reported for the lake and years that independent sources reported.

Of the 250 target lakes, we were able to find verified trophic state data on 93 lakes (38%). For the 93 lakes that had independent lake trophic state data, our models corroborated independent, *in situ* observations 74% of the time, which is consistent with our models’ overall accuracy against testing data from the U.S. EPA NLA. We did not observe any apparent spatial patterns with model misclassification, which complements our spatial confusion validation (Figure S25). Together, these results demonstrate that our manual checking procedure returned similar results as our evaluation procedures against our testing data, giving confidence that our modeling pipeline and evaluation procedures are both robust and able to capture natural processes occurring in lakes.

### Effects of processor heterogeneity

When recreating lake trophic state predictions *de novo*, care should be taken to ensure that effects from heterogeneous processors are minimized. When creating the LTS-US dataset from the original LimnoSat-US dataset^35^, we specified seeds for each modeling framework, which enabled us to reproduce results between model runs. Final dataset production occurred on one machine using an Intel(R) Xeon(R) W-10885M processor with eight cores, however, slight differences may arise due to differences in a user’s hardware float precision.

If users recreate or update LimnoSat-US prior to recreation of the LTS-US predictions, care should be taken as Google Earth Engine^65^ uses a heterogeneous processor framework, where individual processors cannot currently be specified. Meyer *et al*.^9^ quantified the effect of Google Earth Engine’s processor heterogeneity on various lake surface area and basin-level climatological estimations, and effects of processor heterogeneity were likely inconsequential (e.g., differences of 10^−12^), although these differences may result in slightly different trophic state predictions. The extent to which these values would influence results or conclusions of other studies will depend on the level of precision required and scope of research question.

## Usage Notes

The LTS-US dataset was constructed to be an accessible and interoperable product for a range of basic and applied research questions related to water quality and ecological integrity at national scales. Here, we detail several options for application of the LTS-US dataset and associated pipeline.

First, the LTS-US dataset can be joined with water quantity and quality datasets to assess how changes in LTS, and therefore ecosystem integrity, may be influenced by watershed processes, climate, and human population. At the local scale, the LTS-US dataset can be merged with *in situ* sampling data or modeled data from individual lakes to assess how hydrodynamic, climatic, physicochemical, and biological processes may be associated with interannual variation in LTS. As demonstrated here, local *in situ* observations are important for providing validation of the LTS data, and potentially, refinement of methods for deriving LTS predictions. Similarly, the LTS-US dataset can be merged with data from research coordination networks, such as the National Ecological Observatory Network (www.neonscience.org) or the Global Lake Ecological Observatory Network^66^, to enable upscaling highly localized processes to regional and national scales. Beyond watershed-specific processes, the LTS-US dataset can likewise be useful for synthetic questions focused on macroscale water quality trends. For example, in cases where users may wish to synthesize changes in lake ecosystem metabolism with trends in lake water quantity, climate, and human population, the LTS-US dataset can be merged with the GLCP (Global Lake area, Climate, and Population)^9^ or LakeATLAS^67^, thereby enabling users to assess how changes in seasonal and permanent lake surface area may correlate with changes in lake trophic state. The LTS-US dataset offers a valuable resource for addressing a broad spectrum of basic and applied research questions from local and regional to continental scales.

Second, the LTS-US dataset provides a tool for using remote sensing products with the NCP, a framework increasingly used by limnologists, to understand lake water quality at macroscales. Although previous studies have remotely sensed lake trophic state index^68^, our data product is the first to incorporate NCP with remote sensing reflectance data. Where TSI focuses exclusively on eutrophication patterns (also known as greening) associated with nutrient-driven primary production, the LTS-US dataset enables investigations of the spatial extent and temporal trends of lake dystrophication (also known as lake browning). This difference between TSI and NCP is important for assessing long term and spatially extensive changes in lake browning, as well as “murkification” (i.e. simultaneous browning and greening), which has been associated with complex, often non-linear changes in temperature, pH, dissolved oxygen, and food web structure^24,59^. Further, national-scale sampling campaigns, such as the U.S. EPA NLA, have helped reveal that the proportion of dystrophic lakes has been increasing nationally since 2007^24^. The U.S. EPA NLA is one of the most extensive, structured, and coordinated lake sampling efforts at the national scale, and the LTS-US dataset can complement these *in situ* data by providing finer temporal information at comparable spatial scales. When data from successive NLA sampling campaigns become available, the LTS-US dataset can be updated and further benefit from additional training data. Together, the use of remote sensing imagery with extensive sampling campaigns, like the NLA, can be useful for identifying broadscale changes in water quality.

Third, although our reflectance data are spatially aggregated to represent each lake’s characteristic summertime reflectances, our data pipeline and modeling frameworks are amenable to numerous data aggregations, thereby enabling investigation of lakes’ intra- and inter-annual phenologies. For example, many oligotrophic lakes experience summertime greening, due to increased algal growth throughout the summer. Although algal succession tends to follow similar temporal and community compositional patterns^69,70^, users may be interested in understanding how greening events may shift temporally in response to climatic and anthropogenic disturbances. Similarly, end users may be interested in understanding intra-lake heterogeneities, where embayments or nearshore areas may differ in trophic state from the offshore. In both cases, users could adapt our data, modeling, and validation pipeline, where temporal and spatial resolution are more finely resolved. Operationally, end users could modify the aggregation scripts (“1_aggregate.R” and “aggregate_utils.R”)^54^ and LimnoSat codes^35^ to accommodate input data that aggregate at monthly or fortnightly timesteps as well as on a per-pixel basis or with varying radii lengths from the Chebyshev center. Because our data pipeline allows for automated re-running of all harmonization, modeling, and quality control routines, users are able to build off the existing infrastructure to tailor the LTS-US dataset to their particular research questions without high computational overhead or the need to build new workflows *de novo*.

Beyond any specific research question, the LTS-US dataset is a streamlined resource for many end users looking to incorporate remote sensing and its derived products into their analyses. Because of the dataset’s interoperability and flexible structure, the LTS-US dataset serves as a powerful resource for evaluating and contextualizing aquatic ecosystem change at local-to-national spatial as well as annual-to-decadal temporal scales.

### Supplementary information

Supplement Tables and Figures

## Data Availability

All data harmonization, modeling, and validation procedures for the LTS-US dataset^54^ were scripted in the R Statistical Environment^71^, using the tidyverse^72^, lubridate^73^, data.table^74^, sf^75^, keras^76^, tensorflow^77^, caret^78^, CAST^79^, yaml^80^, reticulate^81^, xgboost^82^, nnet^47^, viridis^83^, trend^84^, multiROC^85^, ggpubr^86^, fastshap^87^, maps^88^, ggtext^89^, and ggforce^90^ packages. To enhance reproducibility, all scripts are designed to work within a single pipeline that uses the targets package^91^. The targets pipeline is divided into four main components: “1_aggregate”, “2_train”, “3_predict”, and “4_qc”. Each component corresponds to one of the steps presented above and can be customized by users to fit their specific needs. The associated pipeline setup and user guide can be found on the Environmental Data Initiative^54^, where the “README_targets.pdf” file details directory architecture and how to execute the pipeline. When downloading the “scripts.zip” folder to access the targets pipeline, future users should be aware that empty files within the directory are necessary for running the pipeline, as those folders will become populated each time the pipeline is run. To ensure reproducibility across operating platforms, all scripts for the pipeline can be executed within a container. Running the pipeline within the container allows users to execute the entire pipeline without the need to make small, yet important, edits to the code, or to configure their own operating environment to conform to the pipeline’s requirements. For example, recent versions of the sf package default to using the s2 spherical geometry engine instead of the Graphic Environment Operating System (GEOS), which assumes planar coordinates. End users on a system with one version of the sf library might need to adjust the code to use the correct geometry engine, whereas users with another version might be able to run the pipeline without any adjustments. The container crystallizes a known-working set of libraries, both at the system level (e.g., GEOS, GDAL, PROJ) and at the R level (e.g., sf), so that anybody can run the code without reconfiguring their own environment. This also provides future proofing by ensuring that the inevitable changes to other libraries over time do not lead to errors. To help end users, who are less familiar with running containerized code, a tutorial for installing and executing the pipeline within the container is located in the Environmental Data Initiative repository as a compressed entity (see “README_container.pdf”)^54^. The EDI repository also contains both a rendered (“lake_trophic_status_docker_image.tar.gz”; ~3.5 GB) and unrendered (“lts_container.zip”; ~4.0 KB) docker image. While the document “README_container.pdf” details information for running both the rendered and unrendered images, future users can choose either format depending on their familiarity with rendering Docker images and their capacity to download larger Docker images.

## References

[1] Mekonnen MM, Hoekstra AY (2016). Four billion people facing severe water scarcity. Science Advances.

[2] Topp SN (2021). Shifting Patterns of Summer Lake Color Phenology in Over 26,000 US Lakes. Water Resources Research.

[3] Topp SN (2021). Multi-decadal improvement in US Lake water clarity. Environ. Res. Lett..

[4] Kuhn C, Butman D (2021). Declining greenness in Arctic-boreal lakes. Proceedings of the National Academy of Sciences.

[5] Paltsev A, Creed IF (2022). Are Northern Lakes in Relatively Intact Temperate Forests Showing Signs of Increasing Phytoplankton Biomass?. Ecosystems.

[6] Zhao G, Li Y, Zhou L, Gao H (2022). Evaporative water loss of 1.42 million global lakes. Nat Commun.

[7] Oleksy IA (2022). Heterogenous controls on lake color and trends across the high-elevation U.S. Rocky Mountain region. Environ. Res. Lett..

[8] Pekel J-F, Cottam A, Gorelick N, Belward AS (2016). High-resolution mapping of global surface water and its long-term changes. Nature.

[9] Meyer MF, Labou SG, Cramer AN, Brousil MR, Luff BT (2020). The global lake area, climate, and population dataset. Sci Data.

[10] Khandelwal A (2022). ReaLSAT, a global dataset of reservoir and lake surface area variations. Sci Data.

[11] Carrea L (2023). Satellite-derived multivariate world-wide lake physical variable timeseries for climate studies. Sci Data.

[12] Gardner JR (2021). The Color of Rivers. Geophysical Research Letters.

[13] Yang X (2022). The Color of Earth’s Lakes. Geophysical Research Letters.

[14] Kraemer BM, Kakouei K, Munteanu C, Thayne MW, Adrian R (2022). Worldwide moderate-resolution mapping of lake surface chl-a reveals variable responses to global change (1997–2020). PLOS Water.

[15] Hou X (2022). Global mapping reveals increase in lacustrine algal blooms over the past decade. Nat. Geosci..

[16] Read EK (2017). Water quality data for national-scale aquatic research: The Water Quality Portal. Water Resources Research.

[17] Ross MRV (2019). AquaSat: A Data Set to Enable Remote Sensing of Water Quality for Inland Waters. Water Resources Research.

[18] Wetzel, R. G. *Limnology: Lake and River Ecosystems*. (Academic Press, 2001).

[19] USEPA. *The National Eutrophication Survey*. (1972).

[20] Ledesma JLJ, Köhler SJ, Futter MN (2012). Long-term dynamics of dissolved organic carbon: Implications for drinking water supply. Science of The Total Environment.

[21] Carlson RE (1977). A trophic state index for lakes. Limnology and Oceanography.

[22] Williamson CE, Morris DP, Pace ML, Olson OG (1999). Dissolved organic carbon and nutrients as regulators of lake ecosystems: Resurrection of a more integrated paradigm. Limnology and Oceanography.

[23] Webster KE (2008). An empirical evaluation of the nutrient-color paradigm for lakes. Limnology and Oceanography.

[24] Leech DM, Pollard AI, Labou SG, Hampton SE (2018). Fewer blue lakes and more murky lakes across the continental U.S.: Implications for planktonic food webs. Limnology and Oceanography.

[25] USEPA. *Survey of the Nation’s Lakes. Field Operations Manual* (2007).

[26] USEPA. *2012 National Lakes Assessment. Field Operations Manual.*, (2011).

[27] USEPA. *National Lakes Assessment. Laboratory Operations Manual*. (2012).

[28] USEPA. *National Lakes Assessment 2017. Field Operations Manual*. (2017).

[29] USEPA. *National Lakes Assessment 2017. Laboratory Operations Manual. V.1.1*. (2017).

[30] Omernik JM (1987). Ecoregions of the Conterminous United States. Annals of the Association of American Geographers.

[31] USEPA. *Handbook of Methods for Acid Deposition Studies: Laboratory Analyses for Surface Water Chemistry*. (U.S. Environmental Protection Agency, Office of Research and Development, 1987).

[32] APHA. *Standard Methods for the Examination of Water and Wastewater. American Public Health Association, Washington DC*. (American Public Health Association, 1999).

[33] Messager ML, Lehner B, Grill G, Nedeva I, Schmitt O (2016). Estimating the volume and age of water stored in global lakes using a geo-statistical approach. Nat Commun.

[34] Robinson N, Regetz J, Guralnick RP (2014). EarthEnv-DEM90: A nearly-global, void-free, multi-scale smoothed, 90m digital elevation model from fused ASTER and SRTM data. ISPRS Journal of Photogrammetry and Remote Sensing.

[35] Topp S, Pavelsky T, Yang X, Gardner J, Ross MRV (2020). LimnoSat-US: A Remote Sensing Dataset for U.S. Lakes from 1984–2020..

[36] Shen Z, Yu X, Sheng Y, Li J, Luo J (2015). A Fast Algorithm to Estimate the Deepest Points of Lakes for Regional Lake Registration. PLOS ONE.

[37] Jones JW (2019). Improved Automated Detection of Subpixel-Scale Inundation—Revised Dynamic Surface Water Extent (DSWE) Partial Surface Water Tests. Remote Sensing.

[38] Foga S (2017). Cloud detection algorithm comparison and validation for operational Landsat data products. Remote Sensing of Environment.

[39] Naumann E (1917). Undersӧkningar ӧver fytoplankton och under den pelagiska regionen fӧsiggående gyttje-och dybildningar inom vissa syd- och mellansvenska urbergsvatten. K. Sv. Vetensk. Akad. Handl..

[40] Thienemann, A. Seetypen. *Naturwissenschaften* **9**, (1921).

[41] Järnefelt H (1925). Zur Limnologie einiger Gewässer Finnlands. Soc. Zool. Bot. Fennicae Vanamo.

[42] Rohde, W. Crystallization of Eutrophication Concepts in Northern Europe. in *Eutrophication: Causes, Consequences, Correctives* 20256. 10.17226/20256 (National Academies Press, 1969).

[43] Nürnberg GK, Shaw M (1998). Productivity of clear and humic lakes: nutrients, phytoplankton, bacteria. Hydrobiologia.

[44] Quinlan R (2021). Relationships of total phosphorus and chlorophyll in lakes worldwide. Limnology and Oceanography.

[45] Paerl HW, Otten TG (2013). Blooms Bite the Hand That Feeds Them. Science.

[46] Downing JA, McCauley E (1992). The nitrogen: phosphorus relationship in lakes. Limnology and Oceanography.

[47] Venables, W. N. & Ripley, B. D. *Modern Applied Statistics with S*. (Springer, 2002).

[48] Friedman JH (2001). Greedy function approximation: A gradient boosting machine. The Annals of Statistics.

[49] Rosenblatt F (1958). The perceptron: A probabilistic model for information storage and organization in the brain. Psychological Review.

[50] Willard JD (2021). Predicting Water Temperature Dynamics of Unmonitored Lakes With Meta-Transfer Learning. Water Resources Research.

[51] Shapley, L. S. 17. A Value for n-Person Games. in *Contributions to the Theory of Games (AM-28), Volume II* (eds. Kuhn, H. W. & Tucker, A. W.) 307–318. 10.1515/9781400881970-018 (Princeton University Press, 1953).

[52] Štrumbelj E, Kononenko I (2014). Explaining prediction models and individual predictions with feature contributions. Knowl Inf Syst.

[53] Lundberg, S. M. & Lee, S.-I. A Unified Approach to Interpreting Model Predictions. in *Advances in Neural Information Processing Systems* (eds. Guyon, I. *et al*.) vol. 30 (Curran Associates, Inc., 2017).

[54] Meyer MF (2023). Environmental Data Initiative.

[55] Langsrud Ø (2003). ANOVA for unbalanced data: Use Type II instead of Type III sums of squares. Statistics and Computing.

[56] Pahlevan N (2021). ACIX-Aqua: A global assessment of atmospheric correction methods for Landsat-8 and Sentinel-2 over lakes, rivers, and coastal waters. Remote Sensing of Environment.

[57] Khazaei B, Read LK, Casali M, Sampson KM, Yates DN (2022). GLOBathy, the global lakes bathymetry dataset. Sci Data.

[58] Vadeboncoeur Y, Peterson G, Zanden MJV, Kalff J (2008). Benthic Algal Production across Lake Size Gradients: Interactions among Morphometry, Nutrients, and Light. Ecology.

[59] Williamson CE (2015). Ecological consequences of long-term browning in lakes. Scientific Reports.

[60] Rosenberger EE, Hampton SE, Fradkin SC, Kennedy BP (2008). Effects of shoreline development on the nearshore environment in large deep oligotrophic lakes. Freshwater Biology.

[61] Hampton SE (2011). Disproportionate importance of nearshore habitat for the food web of a deep oligotrophic lake. Mar. Freshwater Res..

[62] Meyer MF (2022). Effects of spatially heterogeneous lakeside development on nearshore biotic communities in a large, deep, oligotrophic lake. Limnology and Oceanography.

[63] Hampton, S. E. *et al*. Warming-induced changes in benthic redox as a potential driver of increasing benthic algal blooms in high-elevation lakes. *Limnology and Oceanography Letters***n/a**, (2023).

[64] Atkins KS (2022). Integrating periphyton and surface water–groundwater methods to understand lake ecosystem processes. Limnology and Oceanography: Methods.

[65] Gorelick N (2017). Google Earth Engine: Planetary-scale geospatial analysis for everyone. Remote Sensing of Environment.

[66] Weathers KC (2013). The Global Lake Ecological Observatory Network (gleon): The Evolution of Grassroots Network Science. Limnology and Oceanography Bulletin.

[67] Lehner B, Messager ML, Korver MC, Linke S (2022). Global hydro-environmental lake characteristics at high spatial resolution. Sci Data.

[68] Gilarranz LJ, Narwani A, Odermatt D, Siber R, Dakos V (2022). Regime shifts, trends, and variability of lake productivity at a global scale. Proceedings of the National Academy of Sciences.

[69] Sommer U, Gliwicz ZM, Lampert W, Duncan A (1986). The PEG-model of seasonal succession of planktonic events in fresh waters. Archiv für Hydrobiologie.

[70] Sommer U (2012). Beyond the Plankton Ecology Group (PEG) Model: Mechanisms Driving Plankton Succession. Annual Review of Ecology, Evolution, and Systematics.

[71] R Core Team. *R: A Language and Environment for Statistical Computing*. (R Foundation for Statistical Computing, 2022).

[72] Wickham H (2019). Welcome to the tidyverse. Journal of Open Source Software.

[73] Grolemund G, Wickham H (2011). Dates and Times Made Easy with lubridate. Journal of Statistical Software.

[74] Dowle, M. & Srinivasan, A. *data.table: Extension of ‘data.frame’*. (2021).

[75] Pebesma E (2018). Simple Features for R: Standardized Support for Spatial Vector Data. The R Journal.

[76] Allaire, J. J. & Chollet, F. *keras: R Interface to ‘Keras’*. (2022).

[77] Allaire, J. J. & Tang, Y. *tensorflow: R Interface to ‘TensorFlow’*. (2022).

[78] Kuhn, M. *caret: Classification and Regression Training*. (2022).

[79] Meyer, H., Milà, C. & Ludwig, M. *CAST: ‘caret’ Applications for Spatial-Temporal Models*. (2022).

[80] Garbett, S. P. *et al*. *yaml: Methods to Convert R Data to YAML and Back*. (2022).

[81] Ushey, K., Allaire, J. J. & Tang, Y. *reticulate: Interface to ‘Python’*. (2022).

[82] Chen, T. *et al*. *xgboost: Extreme Gradient Boosting*. (2022).

[83] Garnier (2021). viridis - Colorblind-Friendly Color Maps for R..

[84] Pohlert, T. *trend: Non-Parametric Trend Tests and Change-Point Detection*. (2020).

[85] Wei, R. & Wang, J. *multiROC: Calculating and Visualizing ROC and PR Curves Across Multi-Class Classifications*. (2018).

[86] Kassambara, A. *ggpubr: ‘ggplot2’ Based Publication Ready Plots*. (2020).

[87] Greenwell, B. *fastshap: Fast Approximate Shapley Values*. (2021).

[88] Becker, O. S. code by R. A., Minka, A. R. W. R. version by R. B. E. by T. P. & Deckmyn, A. *maps: Draw Geographical Maps*. (2021).

[89] Wilke, C. O. & Wiernik, B. M. *ggtext: Improved Text Rendering Support for ‘ggplot2’*. (2022).

[90] Pedersen, T. L. *ggforce: Accelerating ‘ggplot2’*. (2022).

[91] Landau WM (2021). The targets R package: a dynamic Make-like function-oriented pipeline toolkit for reproducibility and high-performance computing. Journal of Open Source Software.

[92] Oleksy IA, Jones SE, Solomon CT (2021). Hydrologic Setting Dictates the Sensitivity of Ecosystem Metabolism to Climate Variability in Lakes. Ecosystems.

[93] Iannone, R. *DiagrammeR: Graph/Network Visualization*. (2022).

