# Supplementary material for: National-scale remotely sensed lake trophic state from 1984 through 2020
Source: Sci Data. 2024 Jan 16;11:77. doi: 10.1038/s41597-024-02921-0 (PMC10791641; doi:10.1038/s41597-024-02921-0)
Supplement: Supplementary file 1 — Supplement Tables and Figures [file 41597_2024_2921_MOESM1_ESM.docx]

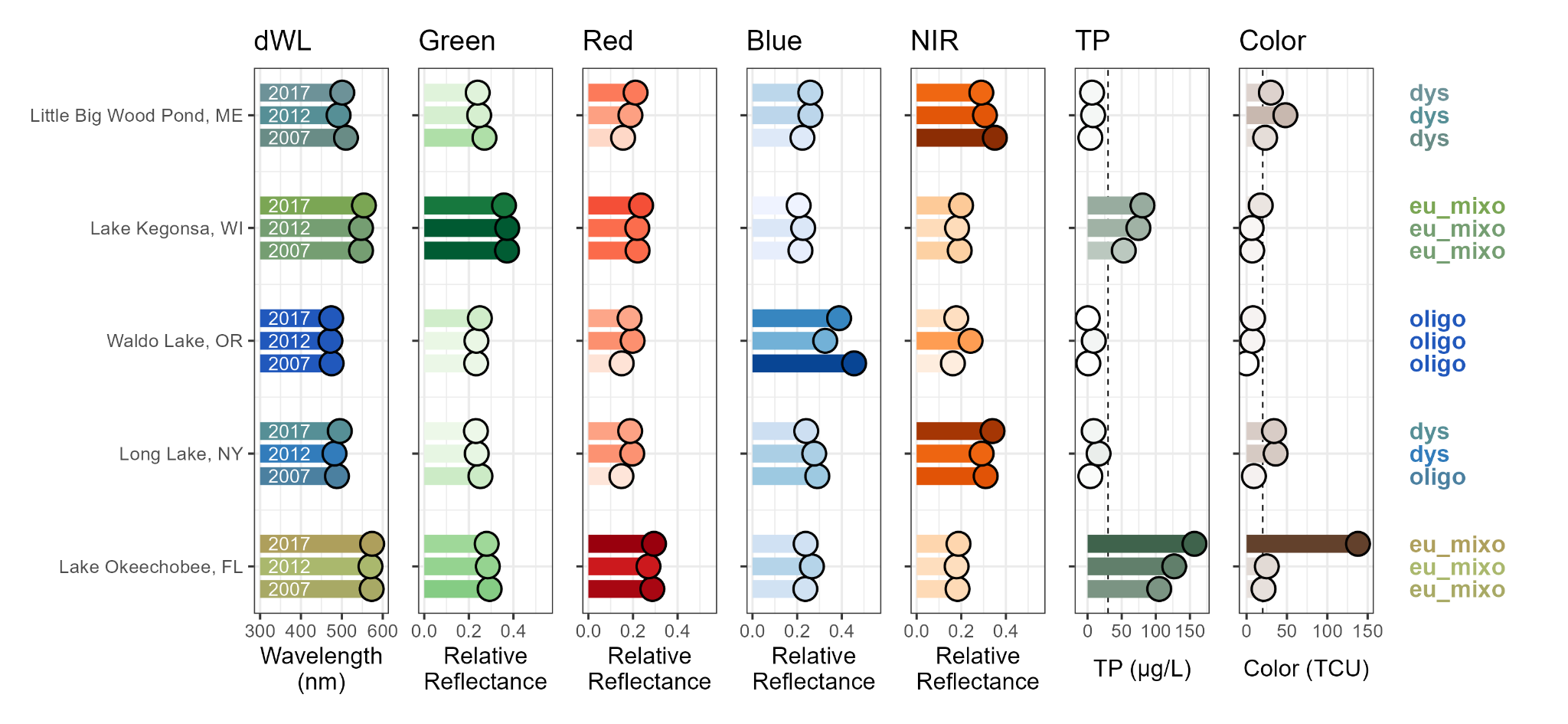


Figure S1: Example comparative summary of five lakes that were sampled in all three U.S. EPA NLA campaigns^24–26^. In general, variation between lakes is visually greater than within a lake^34^. Colors of a lake’s summertime median dominant wavelength (dWL) are represented as the color of the bar and point. All remaining variables are colored by a variable’s value, where a darker bar and point refers to a higher variable value.


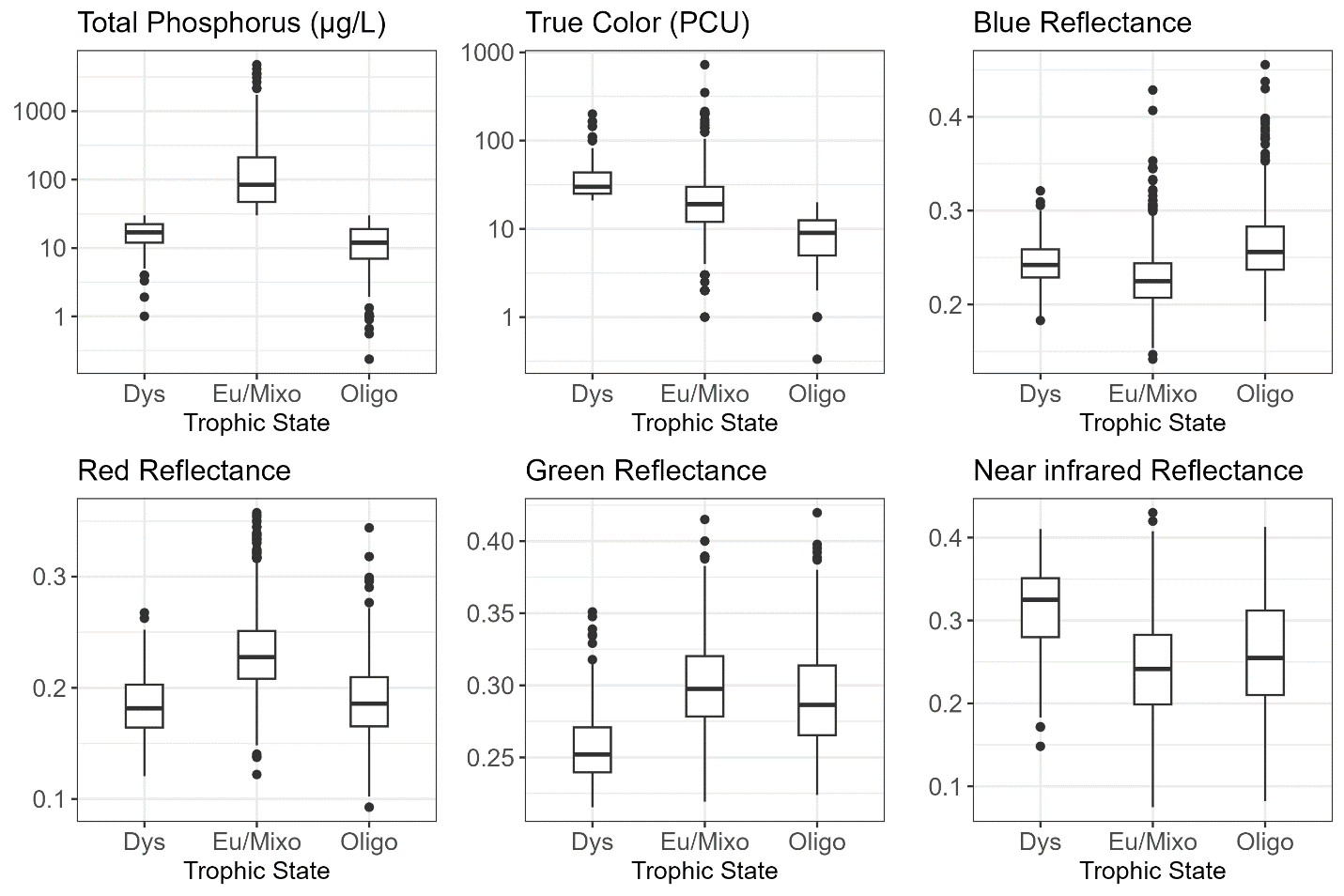


Figure S2: Boxplots of all variables used to define trophic state. Total Phosphorus and True Color data are shown on a log-scale axis to accommodate multiple orders of magnitude.


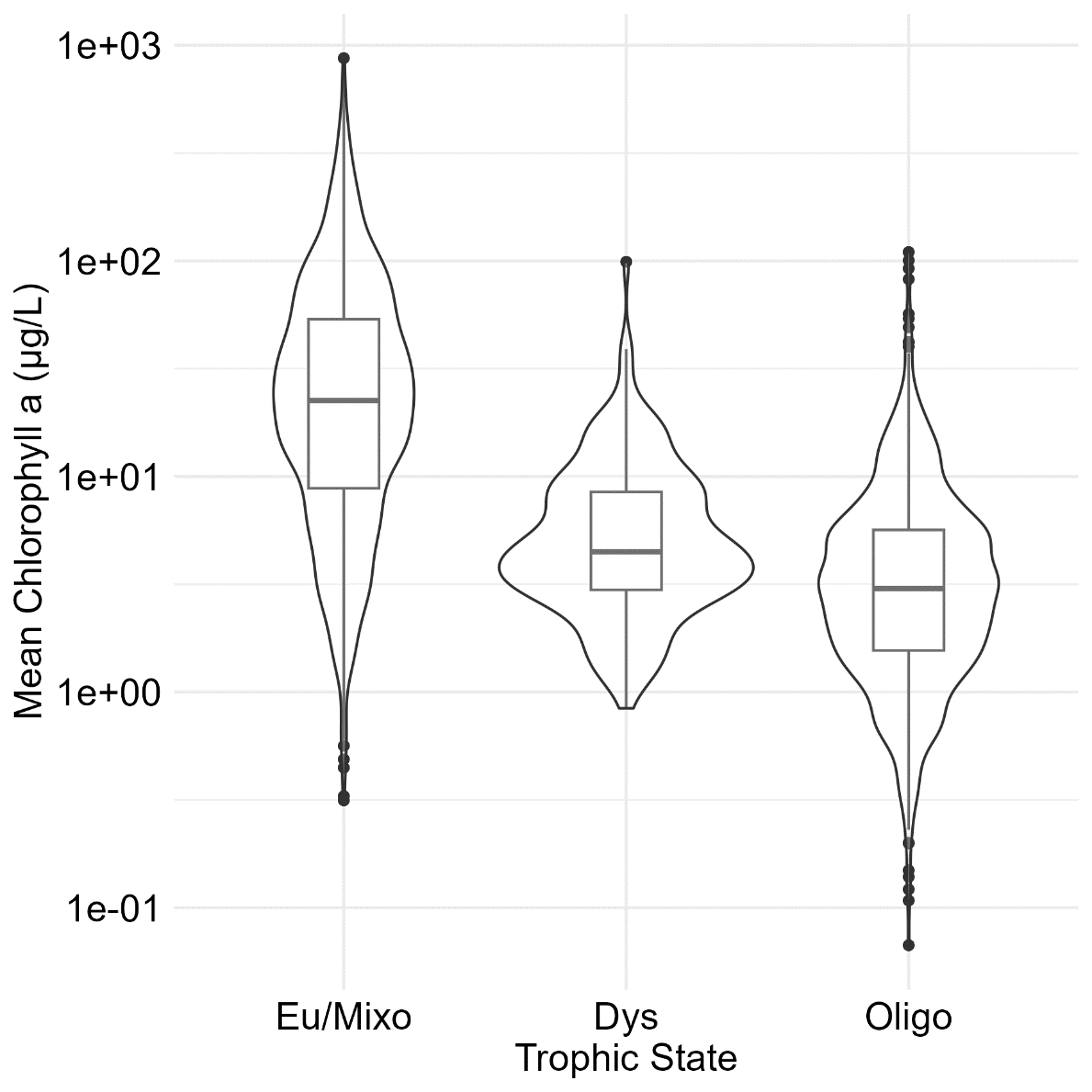


Figure S3: Boxplots and violin plots of summertime chlorophyll a concentrations by lake trophic state. Chlorophyll is shown on a log-transformed axis to compare multiple orders of magnitude.

| Table S1: Summary table of training data used for LTS-US Dataset creation^24–26,34^. These data are used for creating training and test data for each of the three modeling techniques described in the main text. Data are presented as means with standard deviations in parentheses. | | | | | | | | |
| --- | --- | --- | --- | --- | --- | --- | --- | --- |
| Trophic State | Year | Total Phos | Color | Blue | Green | Red | Near Infrared | Number of lakes |
| Dys | 2007 | 16.05 (6.86) | 36.03 (24.12) | 0.24 (0.03) | 0.26 (0.02) | 0.19 (0.03) | 0.31 (0.05) | 40 |
| Dys | 2012 | 19.36 (7.58) | 37.45 (24.21) | 0.24 (0.02) | 0.26 (0.03) | 0.19 (0.04) | 0.31 (0.06) | 62 |
| Dys | 2017 | 15.78 (6.79) | 42.39 (29.96) | 0.25 (0.02) | 0.26 (0.03) | 0.18 (0.02) | 0.32 (0.05) | 81 |
| Eu/Mixo | 2007 | 233.3 (401.7) | 19.87 (14.75) | 0.22 (0.03) | 0.3 (0.03) | 0.23 (0.04) | 0.25 (0.06) | 362 |
| Eu/Mixo | 2012 | 196.61 (362.4) | 26.82 (41.71) | 0.23 (0.03) | 0.3 (0.03) | 0.23 (0.04) | 0.24 (0.06) | 386 |
| Eu/Mixo | 2017 | 169.01 (332.2) | 28.12 (35.53) | 0.23 (0.03) | 0.3 (0.03) | 0.23 (0.03) | 0.24 (0.06) | 252 |
| Oligo | 2007 | 10.85 (7.45) | 8.01 (4.91) | 0.26 (0.04) | 0.29 (0.03) | 0.19 (0.03) | 0.26 (0.07) | 411 |
| Oligo | 2012 | 16.25 (7.5) | 10.89 (4.85) | 0.26 (0.04) | 0.29 (0.04) | 0.18 (0.03) | 0.26 (0.07) | 229 |
| Oligo | 2017 | 14.01 (6.96) | 8.58 (6.03) | 0.27 (0.04) | 0.29 (0.03) | 0.19 (0.03) | 0.26 (0.06) | 224 |


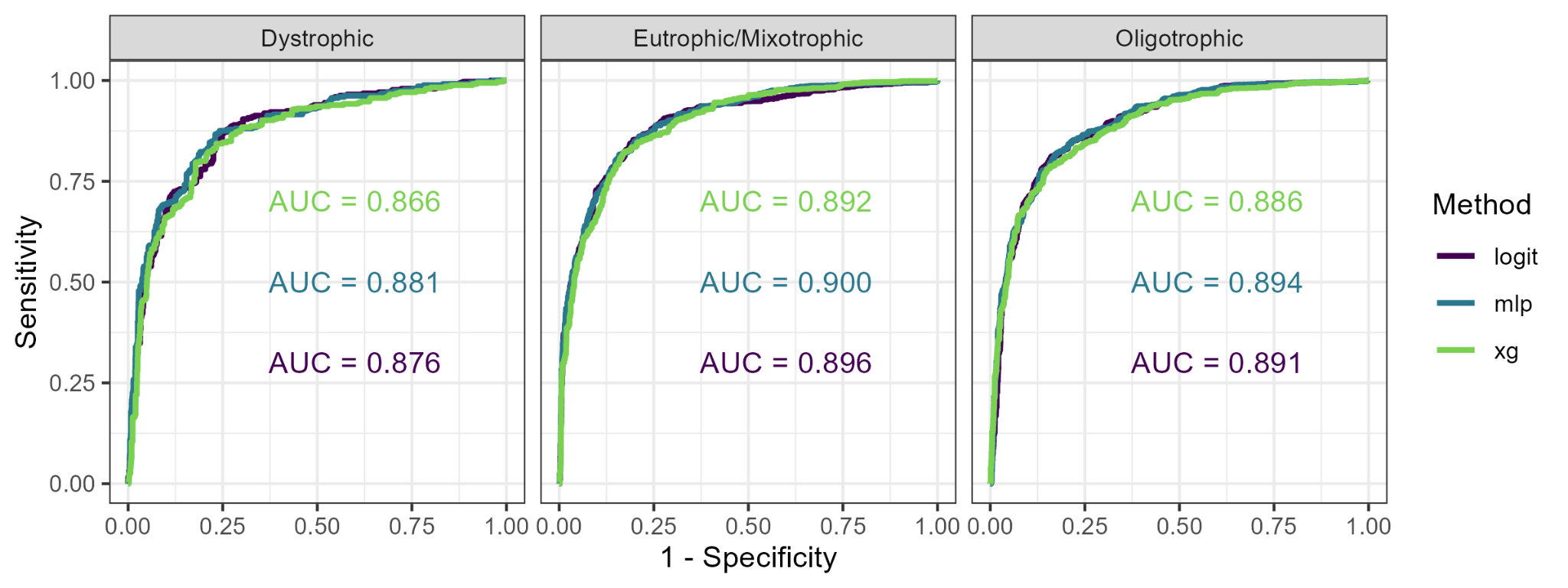


Figure S4: Receiver-Operator-Characteristic (ROC) curves for each trophic status prediction and model method. Area under the Curve (AUC) is reported for each ROC curve. AUC is a metric that generally reflects model fit, where the ROC curve details a model’s capacity to give a true result as the false positive rate is artificially inflated. Across all LTS and modeling methods, ROC curves and resulting AUCs are exceptionally similar, suggesting overall congruence among modeling methodologies.


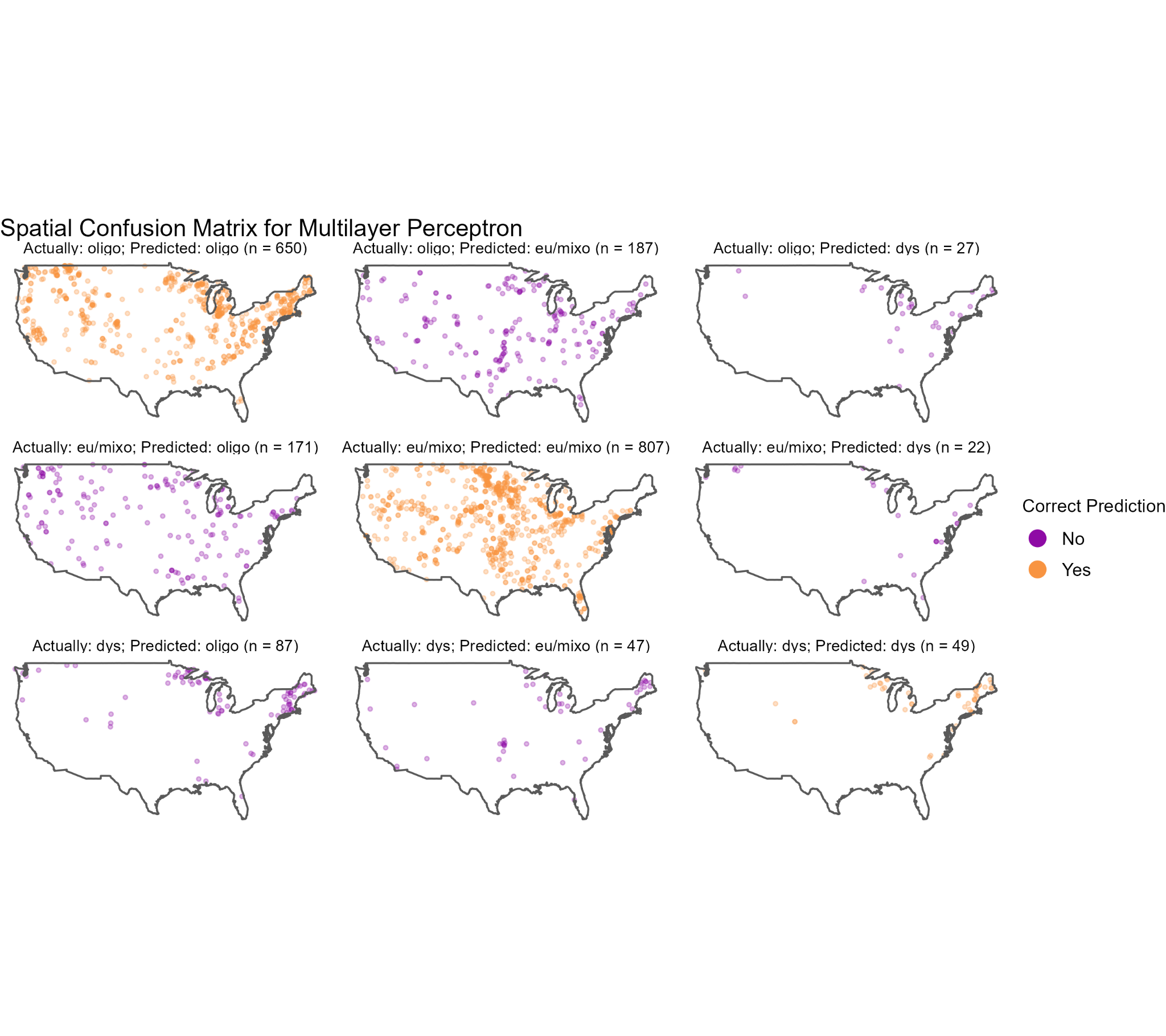


Figure S5: Spatial confusion matrix for multilayer perceptron model. In general, the multilayer perceptron model did not classify or misclassify lakes in a spatial pattern, giving confidence that models were likely misclassifying due to differences other than locational biases at the continental scale.


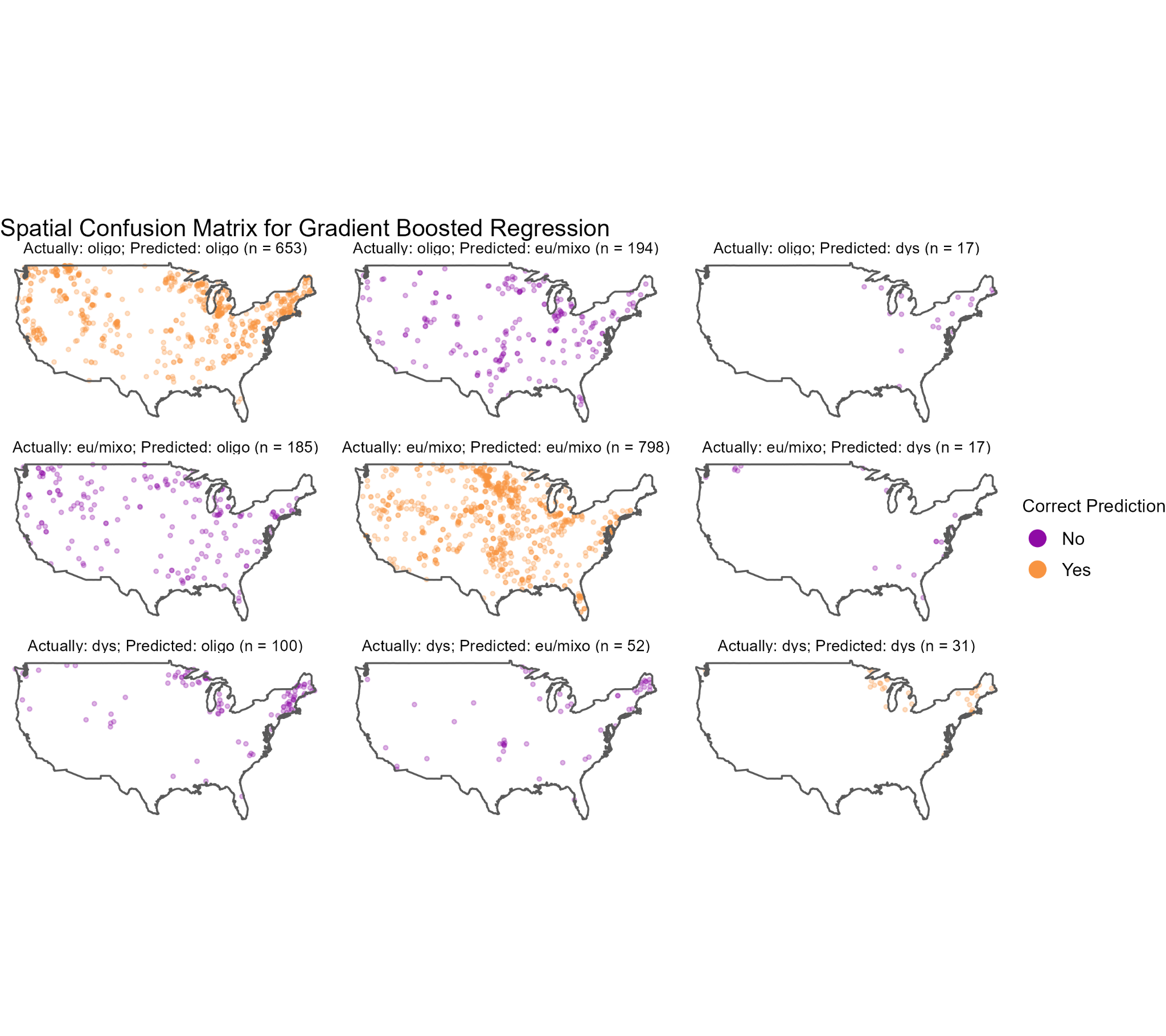


Figure S6: Spatial confusion matrix for gradient boosted regression model. In general, the model did not classify or misclassify lakes in a spatial pattern, giving confidence that models were likely misclassifying due to differences other than locational biases at the continental scale.


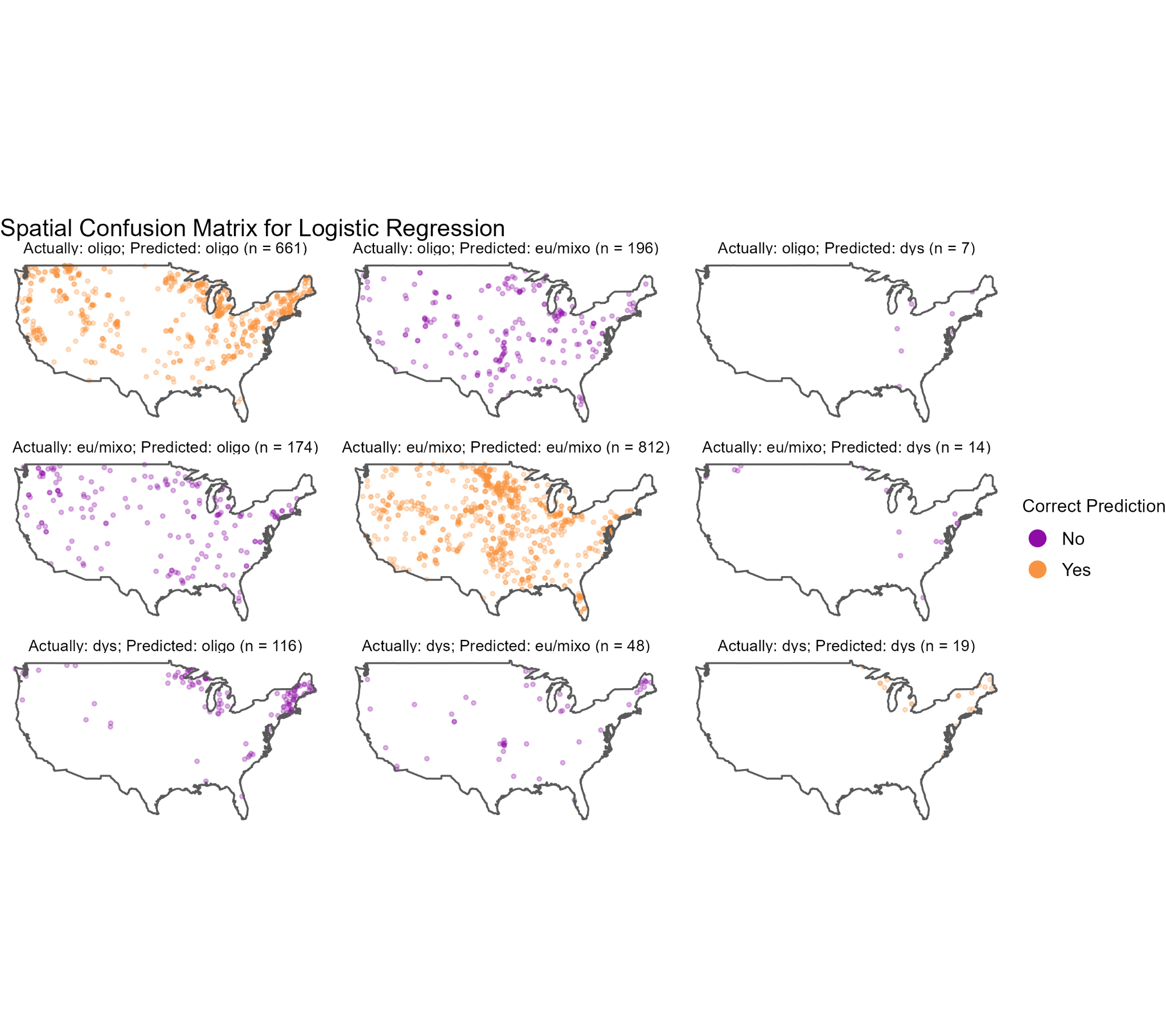
Figure S7: Spatial confusion matrix for Logit model. In general, the Logit model did not classify or misclassify lakes in a spatial pattern, giving confidence that models were likely misclassifying due to differences other than locational biases at the continental scale.


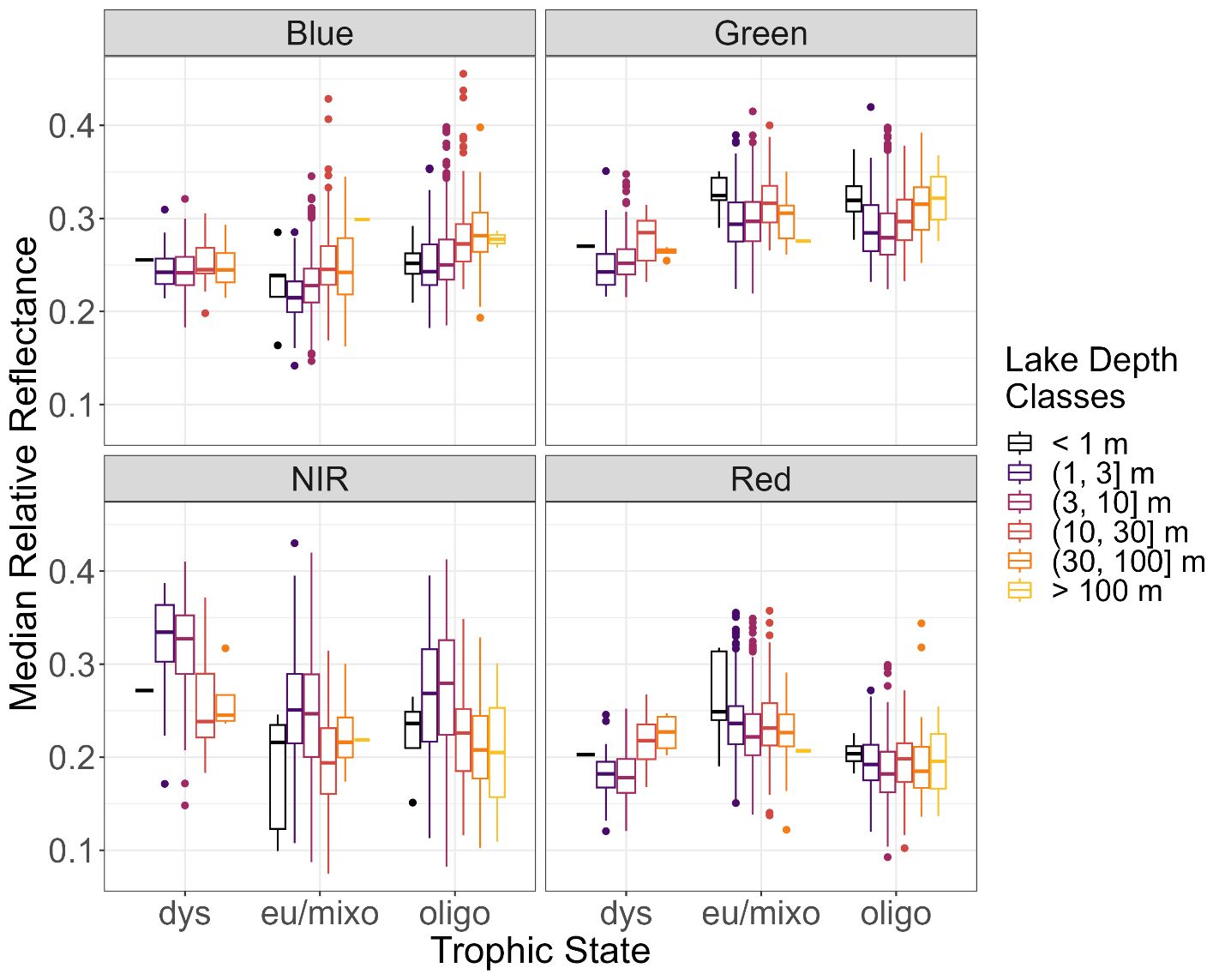


Figure S8: Boxplots of median summertime relative reflectance values for each Landsat band and NCP-defined trophic state divided by lake depth classes. Water quality data are aggregated from the 2007, 2012, and 2017 U.S. EPA NLA campaigns. Reflectance data are aggregated from LimnoSat-US. Relative reflectance is defined as the value of a given band’s reflectance divided by the sum of all four bands. Summertime median relative reflectances are defined as the median of all relative reflectance values from June through August in a given year.


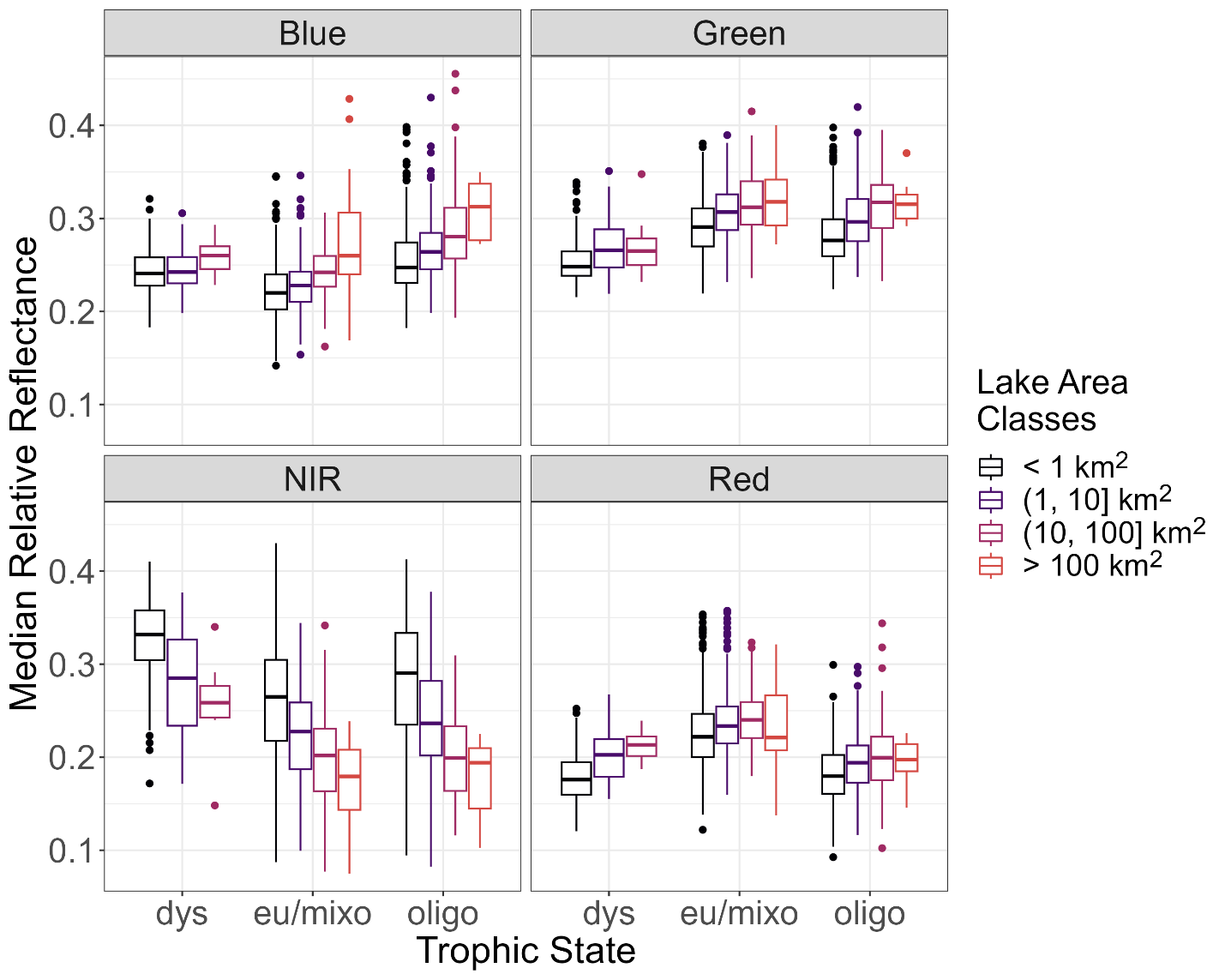


Figure S9: Boxplots of median summertime relative reflectance values for each Landsat band and NCP-defined trophic state divided by lake area classes. Water quality data are aggregated from the 2007, 2012, and 2017 U.S. EPA NLA campaigns. Reflectance data are aggregated from LimnoSat-US. Relative reflectance is defined as the value of a given band’s reflectance divided by the sum of all four bands. Summertime median relative reflectances are defined as the median of all relative reflectance values from June through August in a given year.


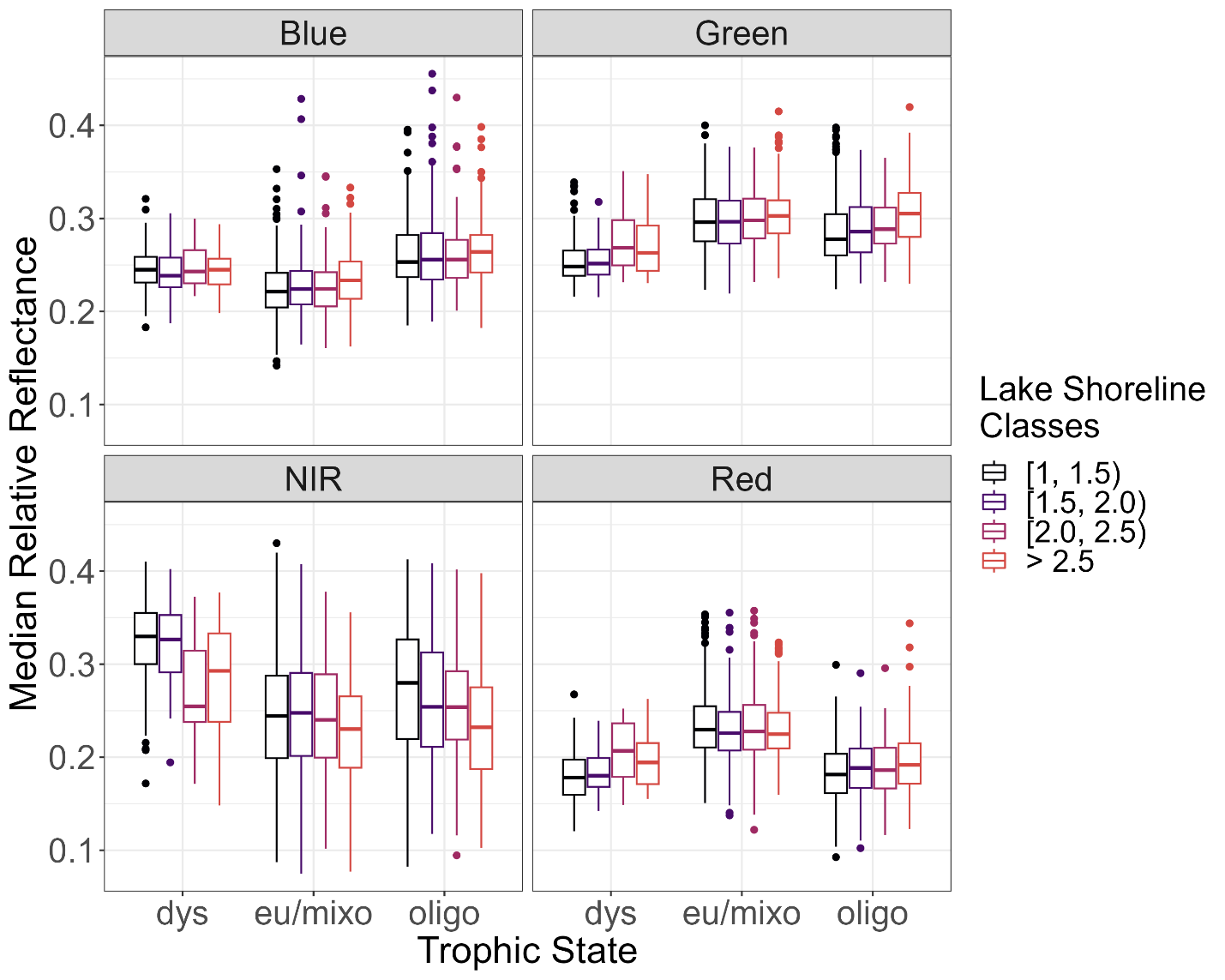


Figure S10: Boxplots of median summertime relative reflectance values for each Landsat band and NCP-defined trophic state divided by lake shoreline development classes. Water quality data are aggregated from the 2007, 2012, and 2017 U.S. EPA NLA campaigns. Reflectance data are aggregated from LimnoSat-US. Relative reflectance is defined as the value of a given band’s reflectance divided by the sum of all four bands. Summertime median relative reflectances are defined as the median of all relative reflectance values from June through August in a given year.


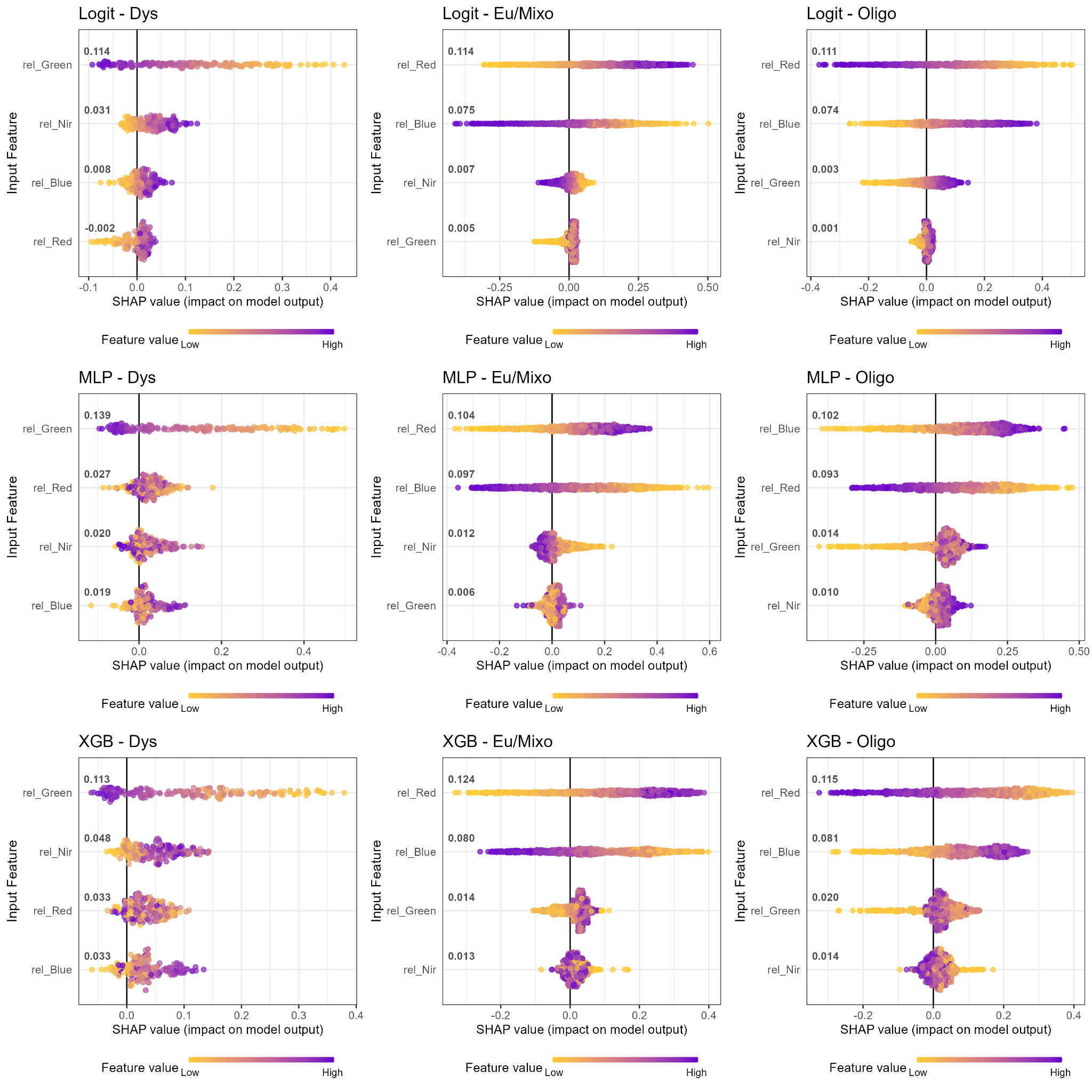


Figure S11: Summary plots from SHAP analysis with SHAP values arranged by model type and lake trophic state. SHAP values include those from correct and incorrect classifications. Importance scores are located next to each feature on the left side of each plot panel. Features are arranged on the y-axis of each plot in order of relative importance, where most important features are at the top of the plot and decrease in relative importance towards the bottom of the plot. Across all modeling types, features were comparable in importance. In all cases, the top two features for each modeling technique and trophic state were identical. Further, the top two features also corresponded to limnological and ecological understanding of each lake type. Dystrophic lakes were most influenced by green and near-infrared bands, which corresponds to these lakes being characterized by increased sediment and dissolved organic carbon as well as decreased primary production. Eutrophic/mixotrophic and oligotrophic lakes were most influenced by red and blue bands, which corresponds to these lakes as being most characterized by primary production.


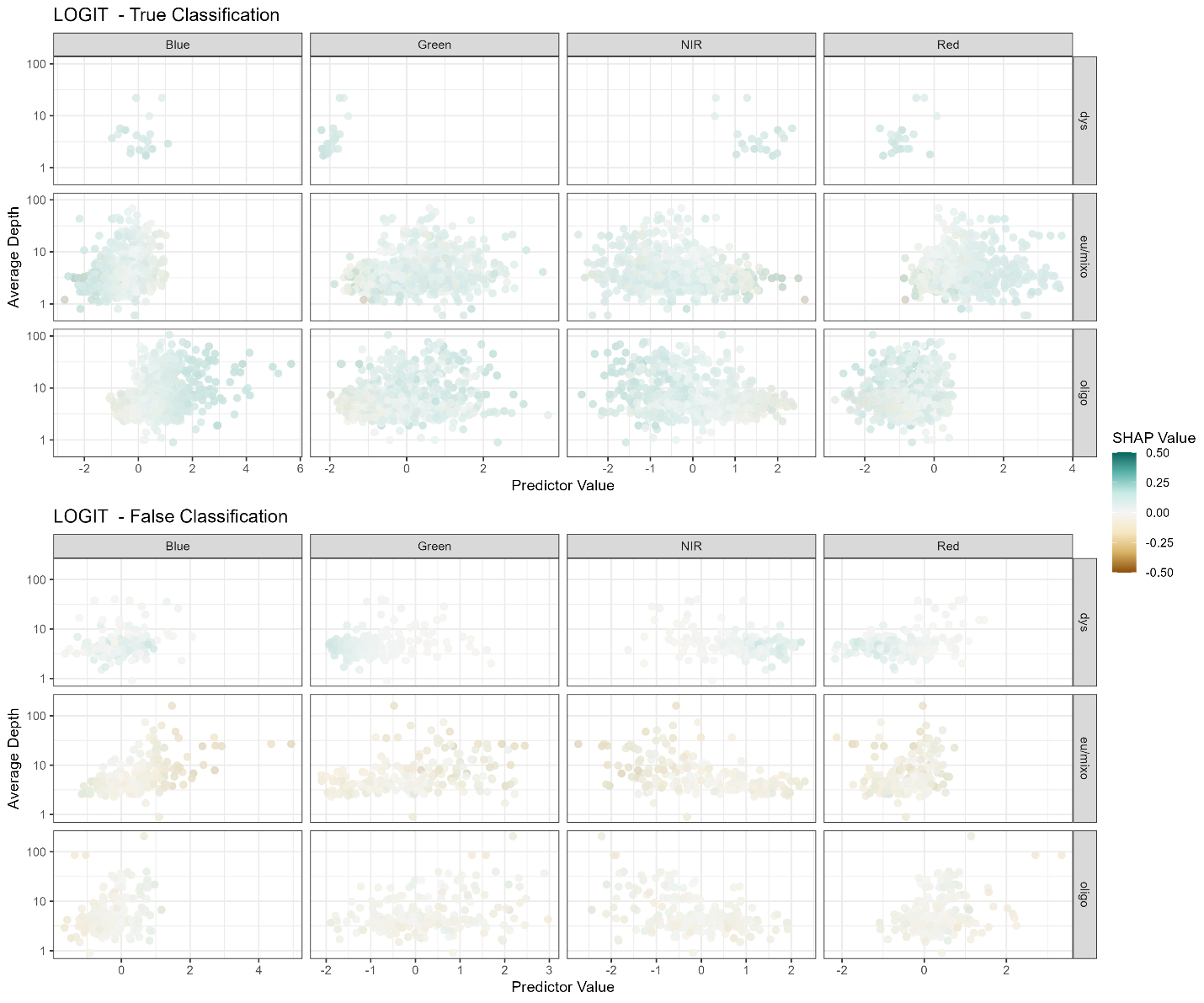


Figure S12: SHAP value analysis by each trophic state’s band value and average depth from logistic regression models. While correct and incorrect classifications generally occupied the same parameter space for band values and average depths, greatest incongruence between correct and incorrect classifications occurred in blue and red bands for eutrophic and oligotrophic lakes. In particular, shallow oligotrophic lakes tended to have lower blue reflectances, which corresponded to a lower SHAP value; shallow eutrophic/mixotrophic lakes likewise had low blue reflectances, but these bands had high SHAP values. Conversely, deeper oligotrophic lakes tended to have lower red band values, which were associated with higher SHAP values; deeper eutrophic/mixotrophic lakes tended to have higher red reflectances, which also had a higher SHAP value. Together, this analysis suggests that lakebed effects may influence classification. For example, benthic algal production in oligotrophic lakes may produce reflectance values similar to eutrophic lakes, leading to model confusion. This same result is implied throughout all analysis steps, where depth appears to be the major issue for correct trophic state classification.


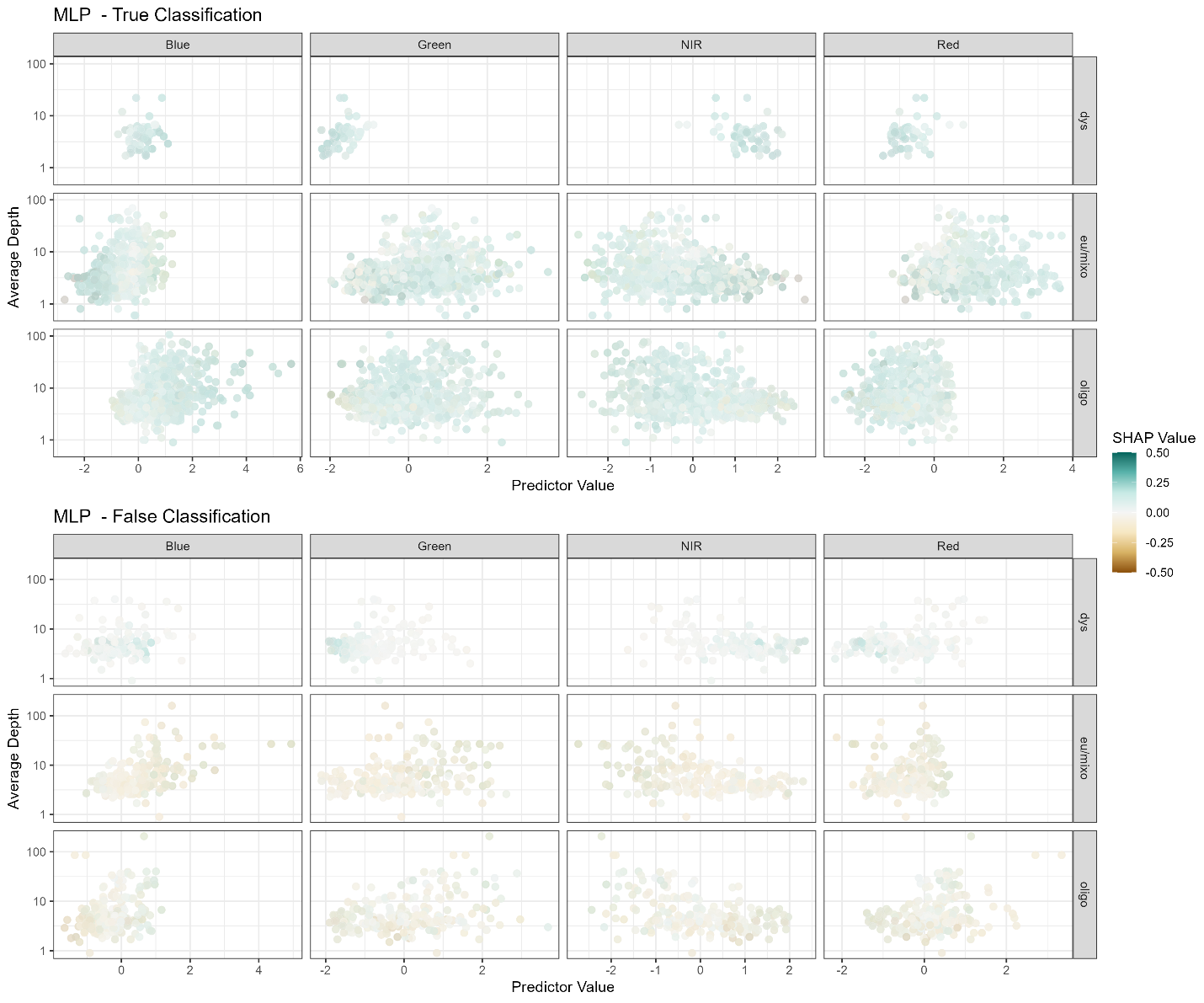


Figure S13: SHAP value analysis by each trophic state’s band value and average depth from multilayer perceptron models. While correct and incorrect classifications generally occupied the same parameter space for band values and average depths, greatest incongruence between correct and incorrect classifications occurred in blue and red bands for eutrophic and oligotrophic lakes. In particular, shallow oligotrophic lakes tended to have lower blue reflectances, which corresponded to a lower SHAP value; shallow eutrophic/mixotrophic lakes likewise had low blue reflectances, but these bands had high SHAP values. Conversely, deeper oligotrophic lakes tended to have lower red band values, which were associated with higher SHAP values; deeper eutrophic/mixotrophic lakes tended to have higher red reflectances, which also had a higher SHAP value. Together, this analysis suggests that lakebed effects may influence classification. For example, benthic algal production in oligotrophic lakes may produce reflectance values similar to eutrophic lakes, leading to model confusion.


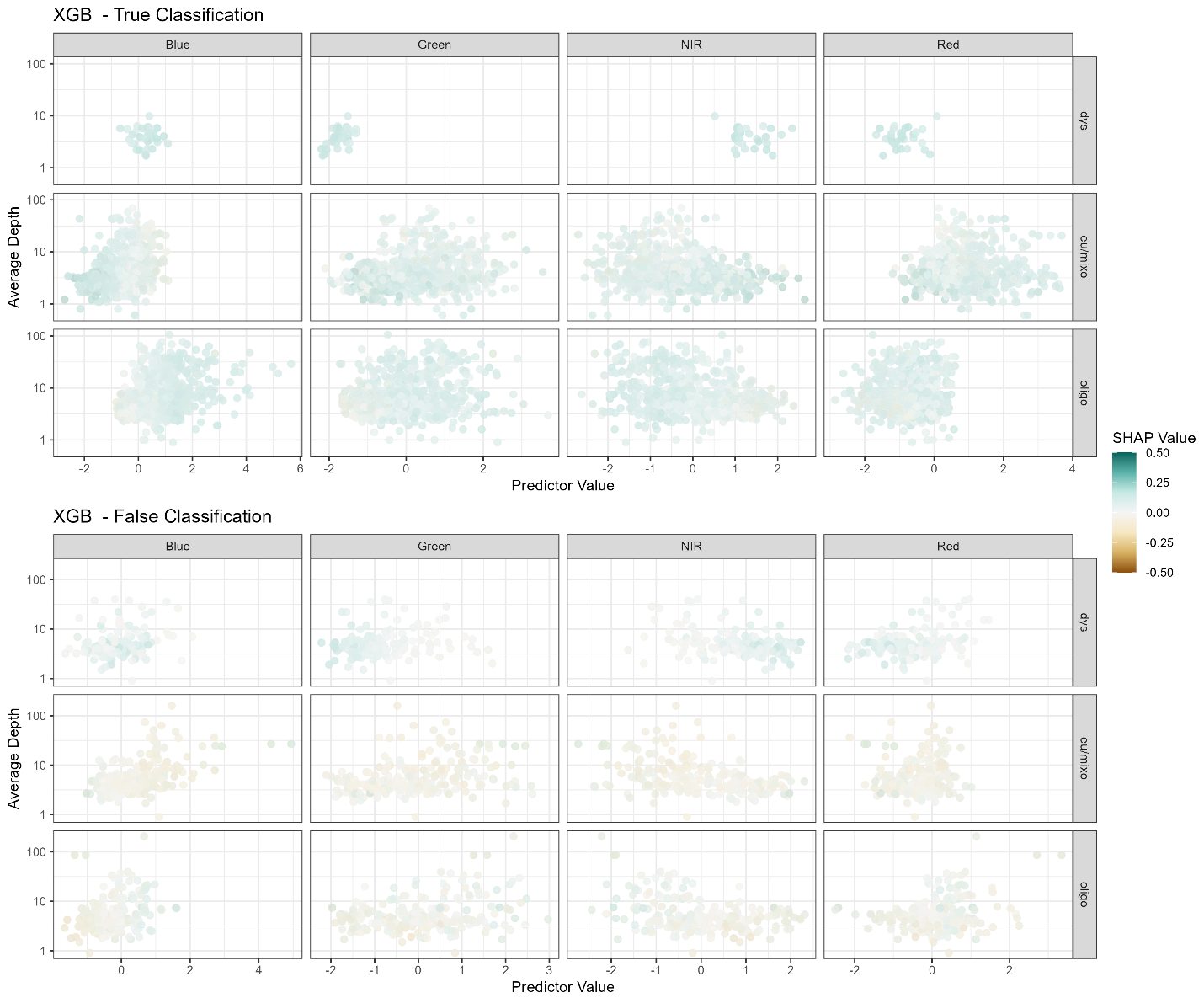


Figure S14: SHAP value analysis by each trophic state’s band value and average depth from gradient boosted regression models. While correct and incorrect classifications generally occupied the same parameter space for band values and average depths, greatest incongruence between correct and incorrect classifications occurred in blue and red bands for eutrophic and oligotrophic lakes. In particular, shallow oligotrophic lakes tended to have lower blue reflectances, which corresponded to a lower SHAP value; shallow eutrophic/mixotrophic lakes likewise had low blue reflectances, but these bands had high SHAP values. Conversely, deeper oligotrophic lakes tended to have lower red band values, which were associated with higher SHAP values; deeper eutrophic/mixotrophic lakes tended to have higher red reflectances, which also had a higher SHAP value. Together, this analysis suggests that lakebed effects may influence classification. For example, benthic algal production in oligotrophic lakes may produce reflectance values similar to eutrophic lakes, leading to model confusion.


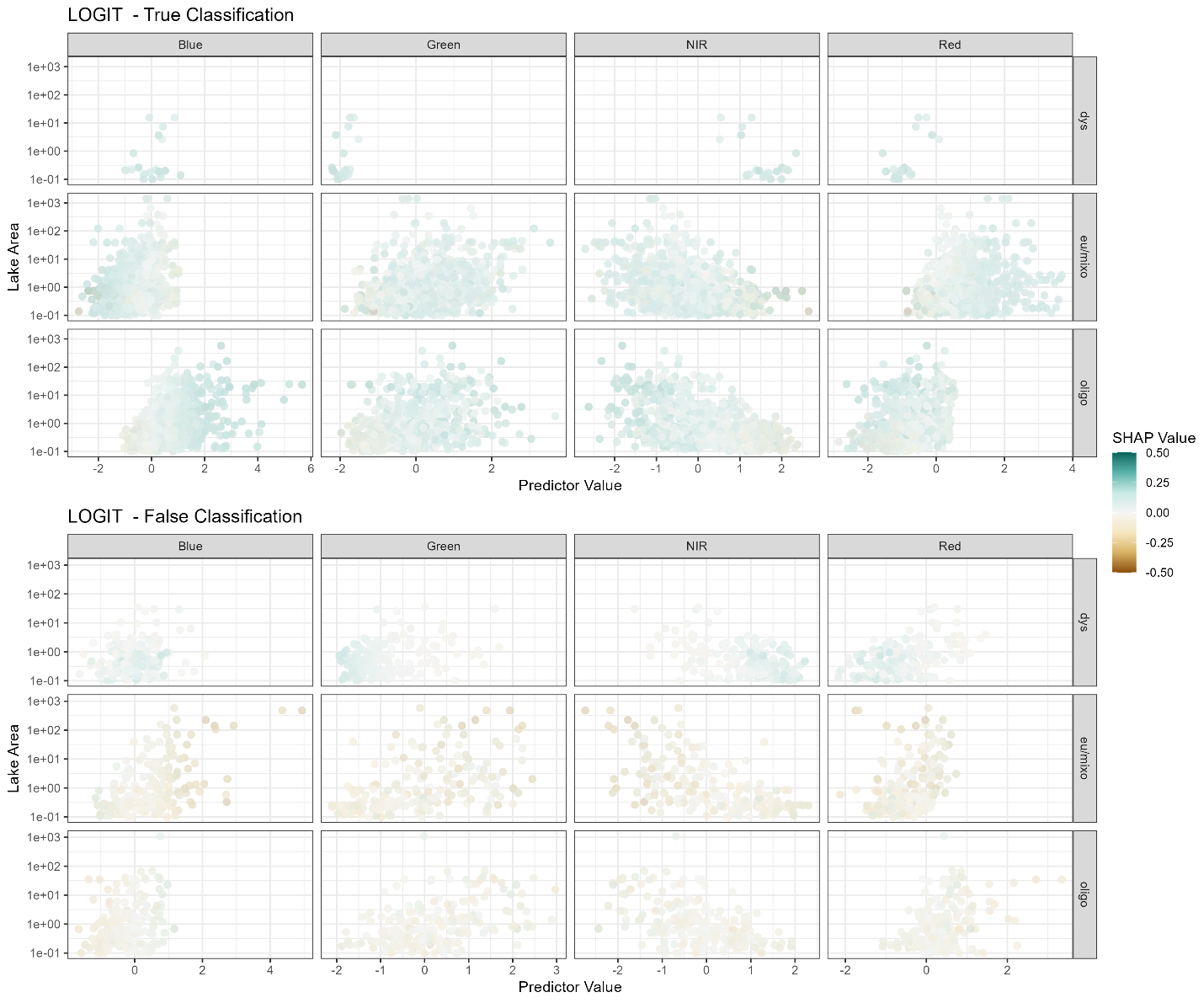


Figure S15: SHAP value analysis by each trophic state’s band value and surface area from logistic regression models. Visually, SHAP and reflectance values as well as lake areas all occupied the same parameter space, implying that lake area, a proxy for adjacency effects, is likely not consequential for feature importance and correct classification. This general result is likewise observed in lake areas being generally consistent across correctly and incorrectly classified lakes.


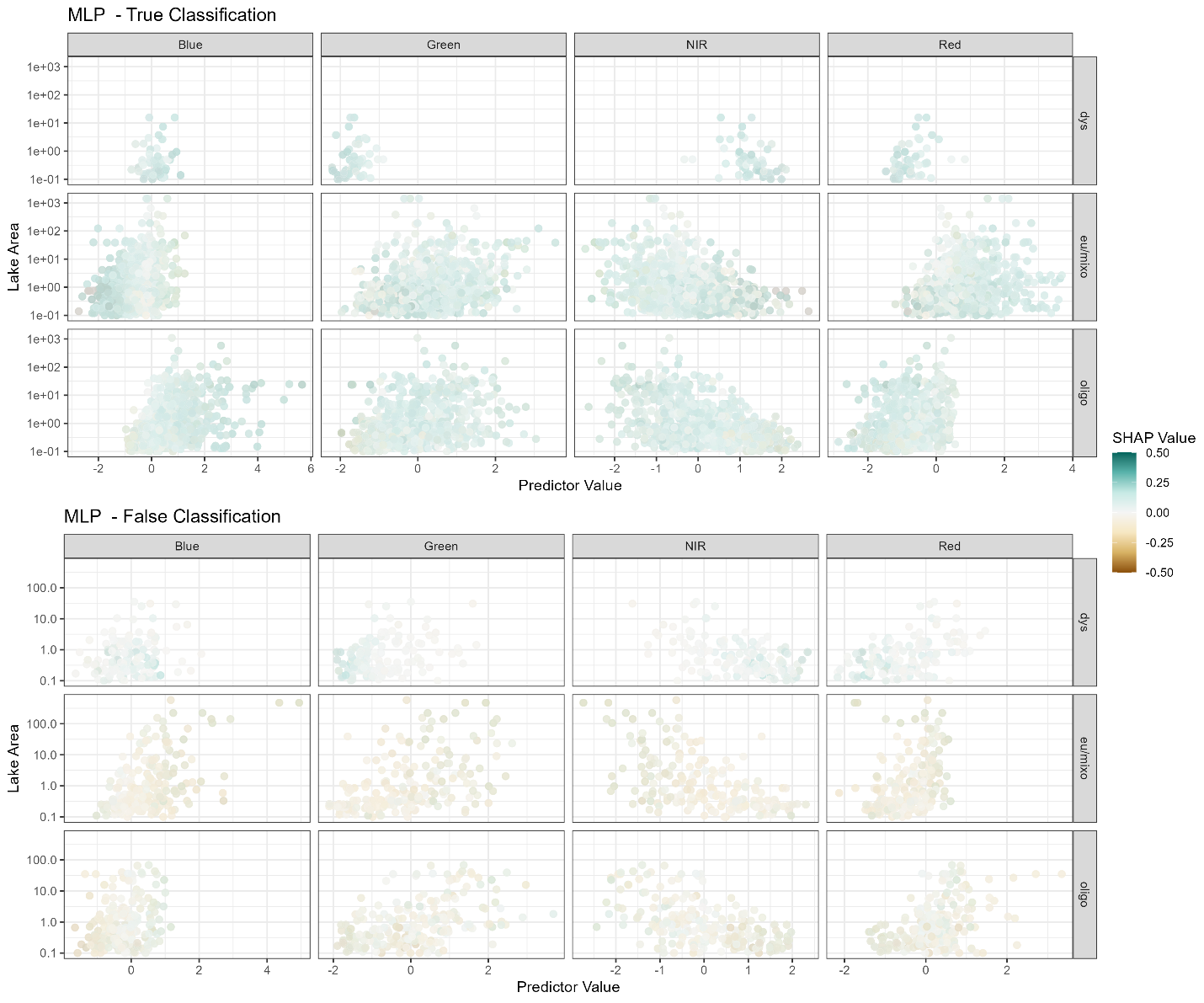


Figure S16: SHAP value analysis by each trophic state’s band value and surface area from multilayer perceptron models. Visually, SHAP and reflectance values as well as lake areas all occupied the same parameter space, implying that lake area, a proxy for adjacency effects, is likely not consequential for feature importance and correct classification. This general result is likewise observed in lake areas being generally consistent across correctly and incorrectly classified lakes.


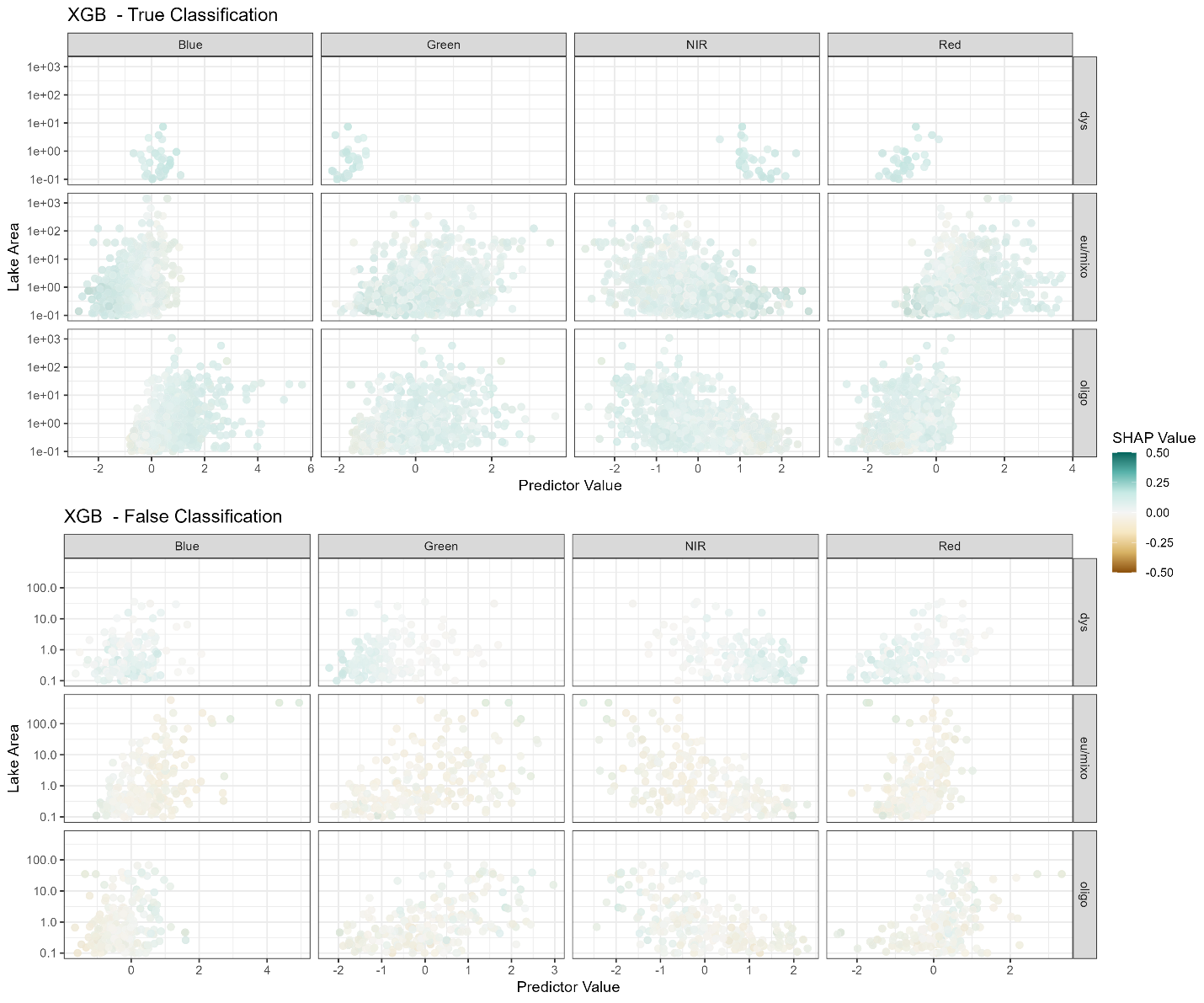


Figure S17: SHAP value analysis by each trophic state’s band value and surface area from gradient boosted regression models. Visually, SHAP and reflectance values as well as lake areas all occupied the same parameter space, implying that lake area, a proxy for adjacency effects, is likely not consequential for feature importance and correct classification. This general result is likewise observed in lake areas being generally consistent across correctly and incorrectly classified lakes.


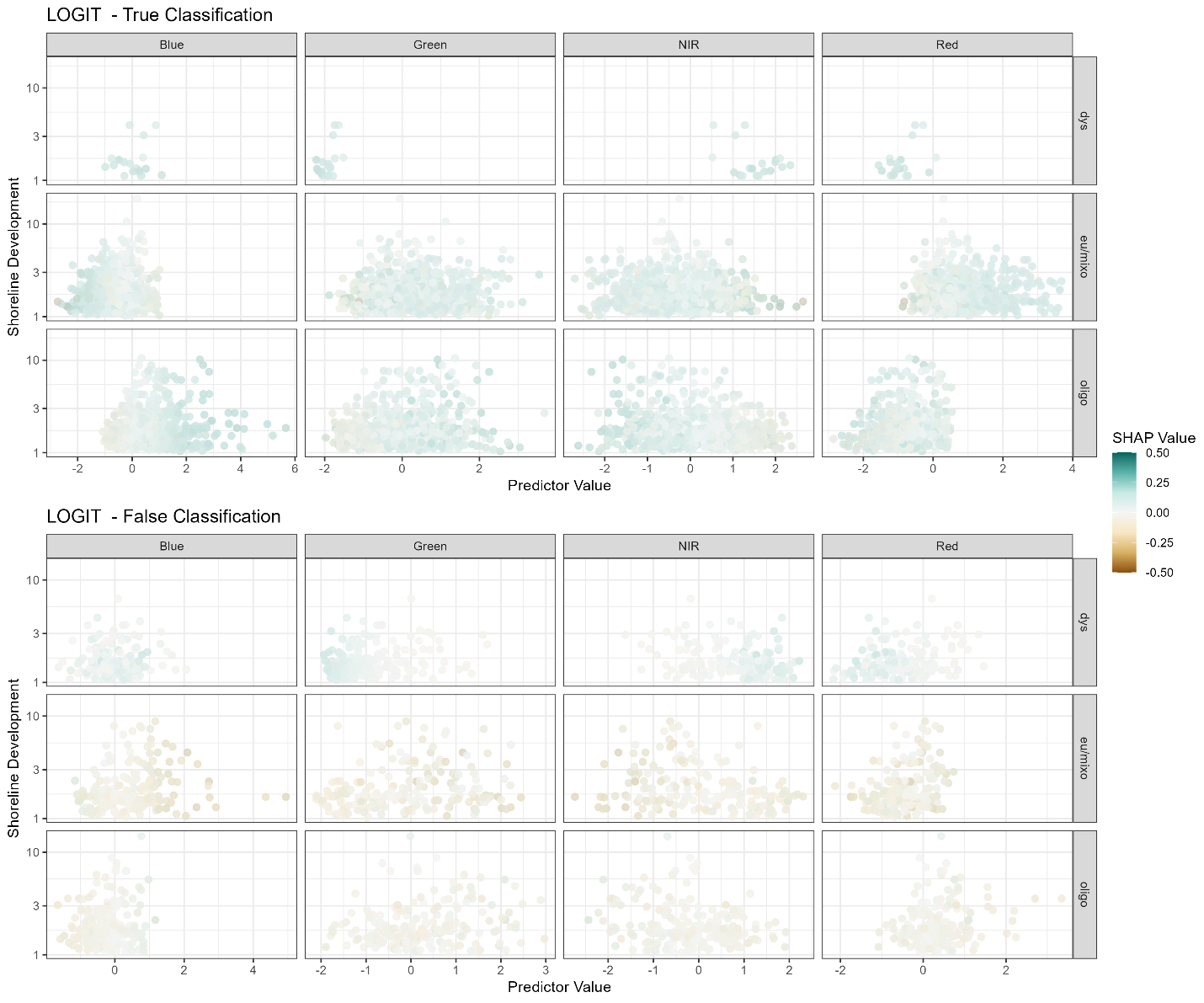


Figure S18: SHAP value analysis by each trophic state’s band value and shoreline development from logistic regression models. Visually, SHAP and reflectance values as well as lake shoreline development all occupied the same parameter space, implying that lake shoreline development, a proxy for adjacency effects, is likely not consequential for feature importance and correct classification. This general result is likewise observed in lake shoreline developments being generally consistent across correctly and incorrectly classified lakes.


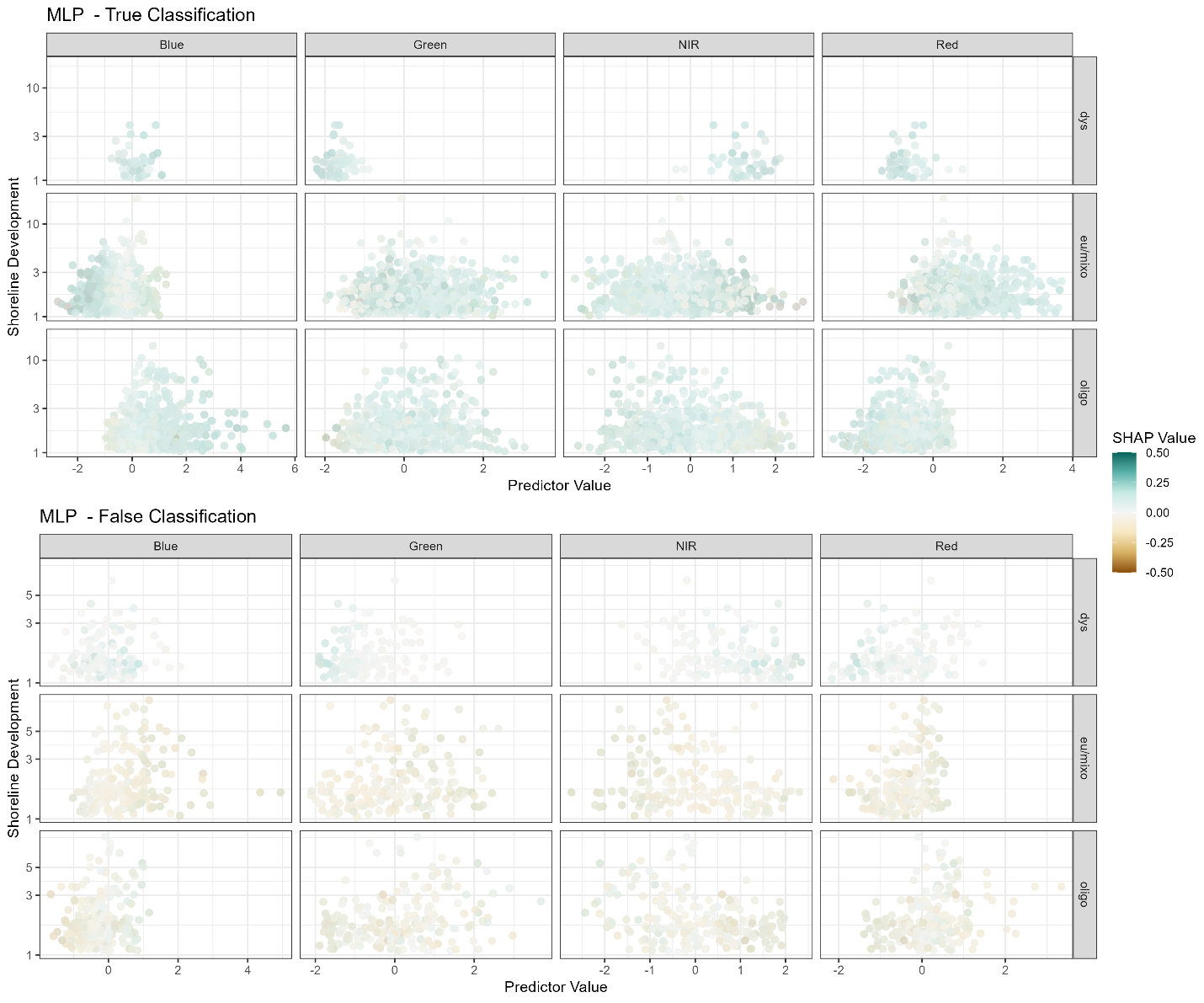


Figure S19: SHAP value analysis by each trophic state’s band value and shoreline development from multilayer perceptron models. Visually, SHAP and reflectance values as well as lake shoreline development all occupied the same parameter space, implying that lake shoreline development, a proxy for adjacency effects, is likely not consequential for feature importance and correct classification. This general result is likewise observed in lake shoreline developments being generally consistent across correctly and incorrectly classified lakes.


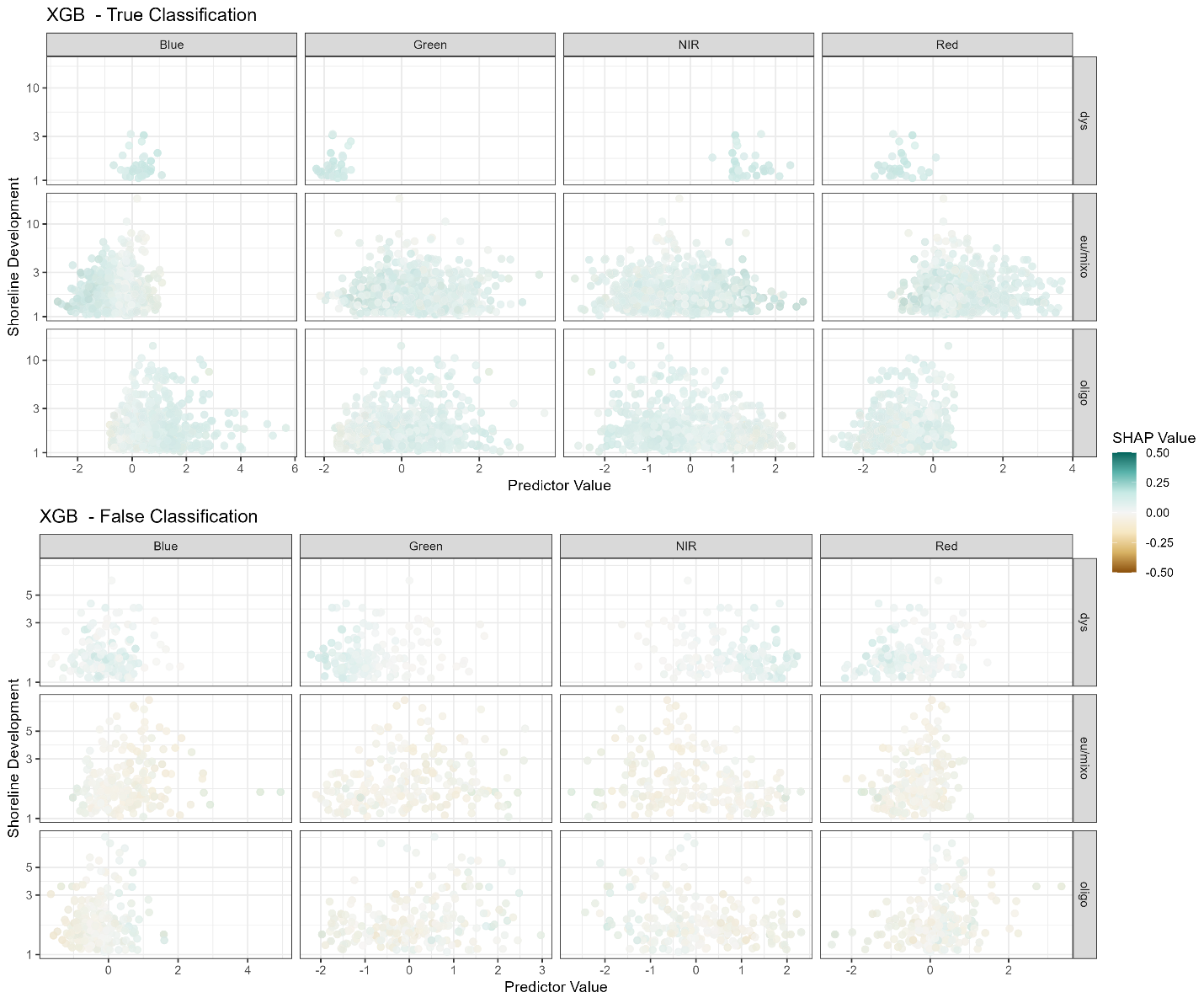


Figure S20: SHAP value analysis by each trophic state’s band value and shoreline development from gradient boosted regression models. Visually, SHAP and reflectance values as well as lake shoreline development all occupied the same parameter space, implying that lake shoreline development, a proxy for adjacency effects, is likely not consequential for feature importance and correct classification. This general result is likewise observed in lake shoreline developments being generally consistent across correctly and incorrectly classified lakes.


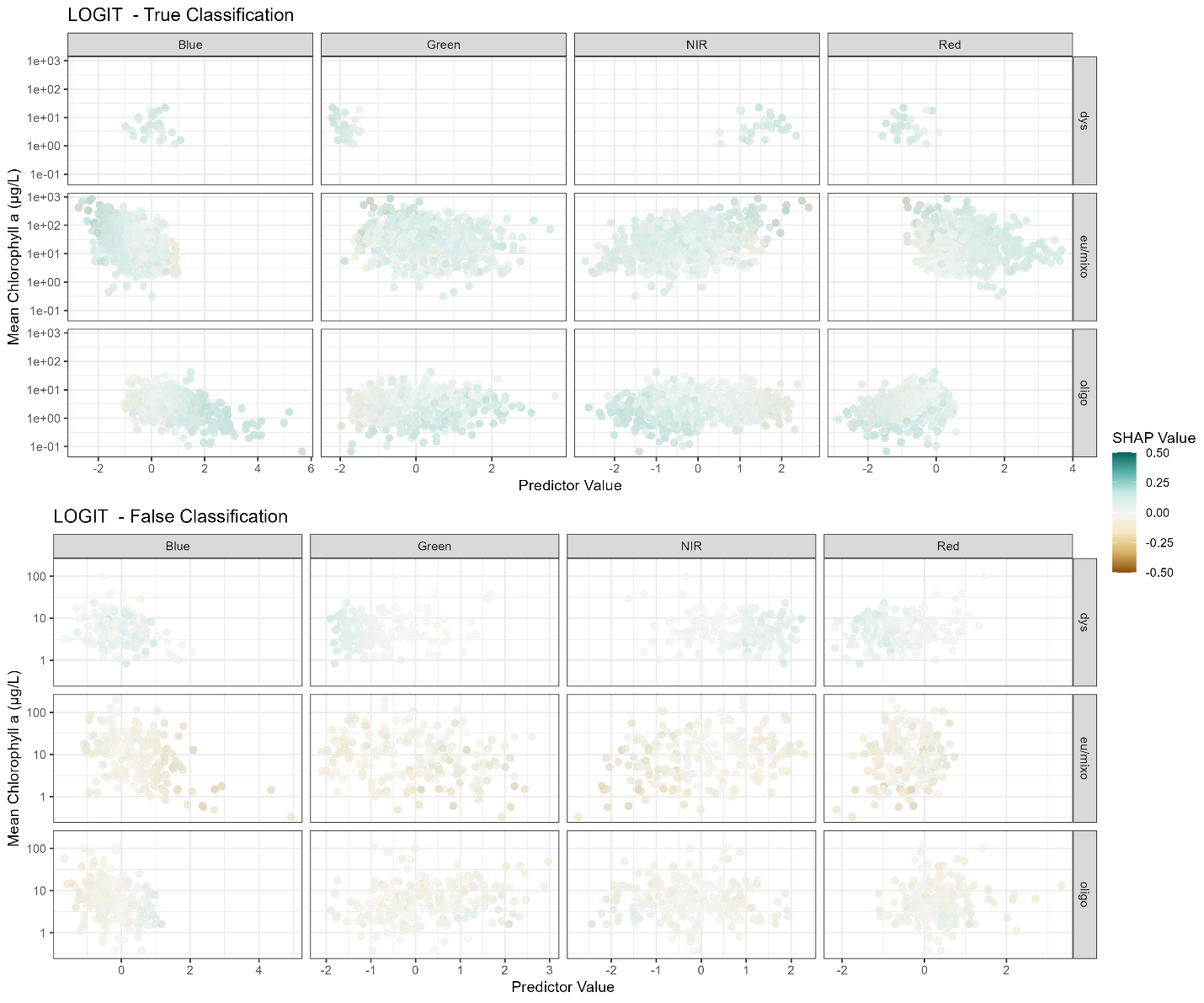


Figure S21: SHAP value analysis by each trophic state’s band value and mean chlorophyll concentration from logistic regression models. Visually, SHAP values for eutrophic/mixotrophic lakes tended to be higher at higher chlorophyll concentrations, whereas high SHAP values for oligotrophic lakes tended to be concentrated at lower chlorophyll concentrations. Trends across spectral band scores were only observed for near-infrared and red band, which like corresponds to these bands conveying information about primary productivity. The general patterns observed across correct and incorrect classification corroborates previous results that misclassifications of LTS most consistently occurs in instances of exceptionally high or low primary productivity for a given lake.


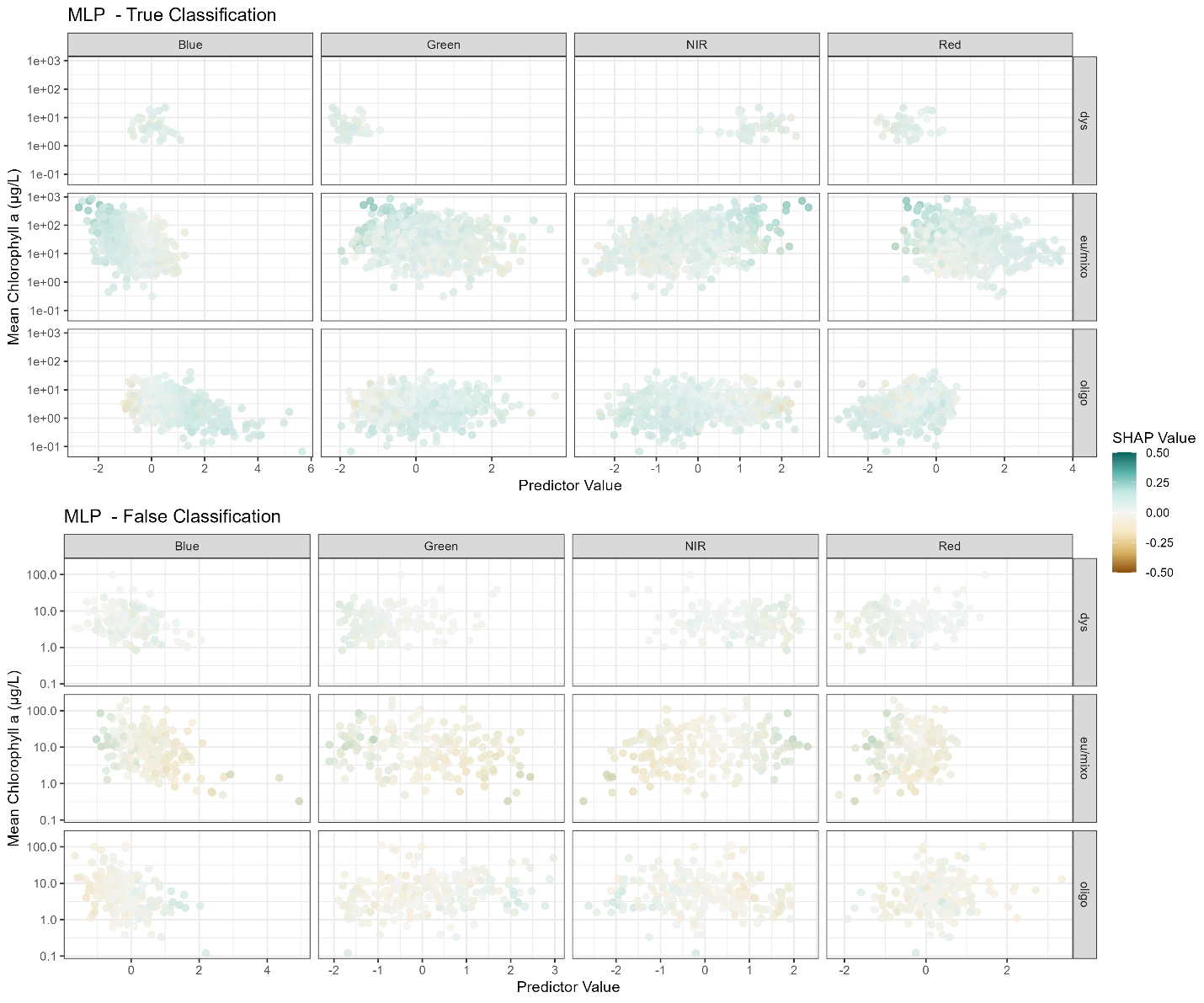


Figure S22: SHAP value analysis by each trophic state’s band value and mean chlorophyll concentration from multilayer perceptron models. Visually, SHAP values for eutrophic/mixotrophic lakes tended to be higher at higher chlorophyll concentrations, whereas high SHAP values for oligotrophic lakes tended to be concentrated at lower chlorophyll concentrations. Trends across spectral band scores were only observed for near-infrared and red band, which like corresponds to these bands conveying information about primary productivity. The general patterns observed across correct and incorrect classification corroborates previous results that misclassifications of LTS most consistently occurs in instances of exceptionally high or low primary productivity for a given lake.


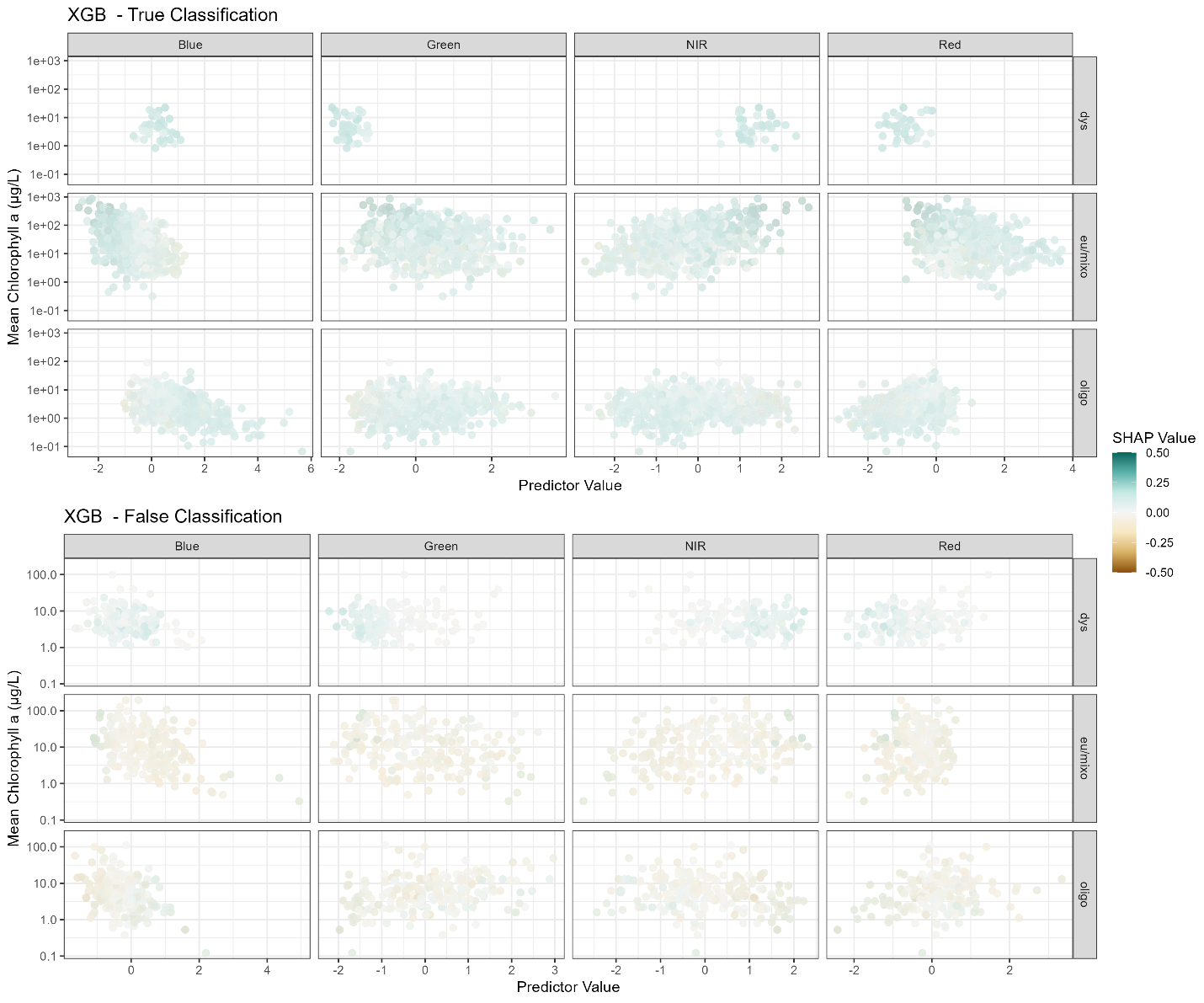


Figure S23: SHAP value analysis by each trophic state’s band value and mean chlorophyll concentration from gradient boosted regression models. Visually, SHAP values for eutrophic/mixotrophic lakes tended to be higher at higher chlorophyll concentrations, whereas high SHAP values for oligotrophic lakes tended to be concentrated at lower chlorophyll concentrations. Trends across spectral band scores were only observed for near-infrared and red band, which like corresponds to these bands conveying information about primary productivity. The general patterns observed across correct and incorrect classification corroborates previous results that misclassifications of LTS most consistently occurs in instances of exceptionally high or low primary productivity for a given lake.


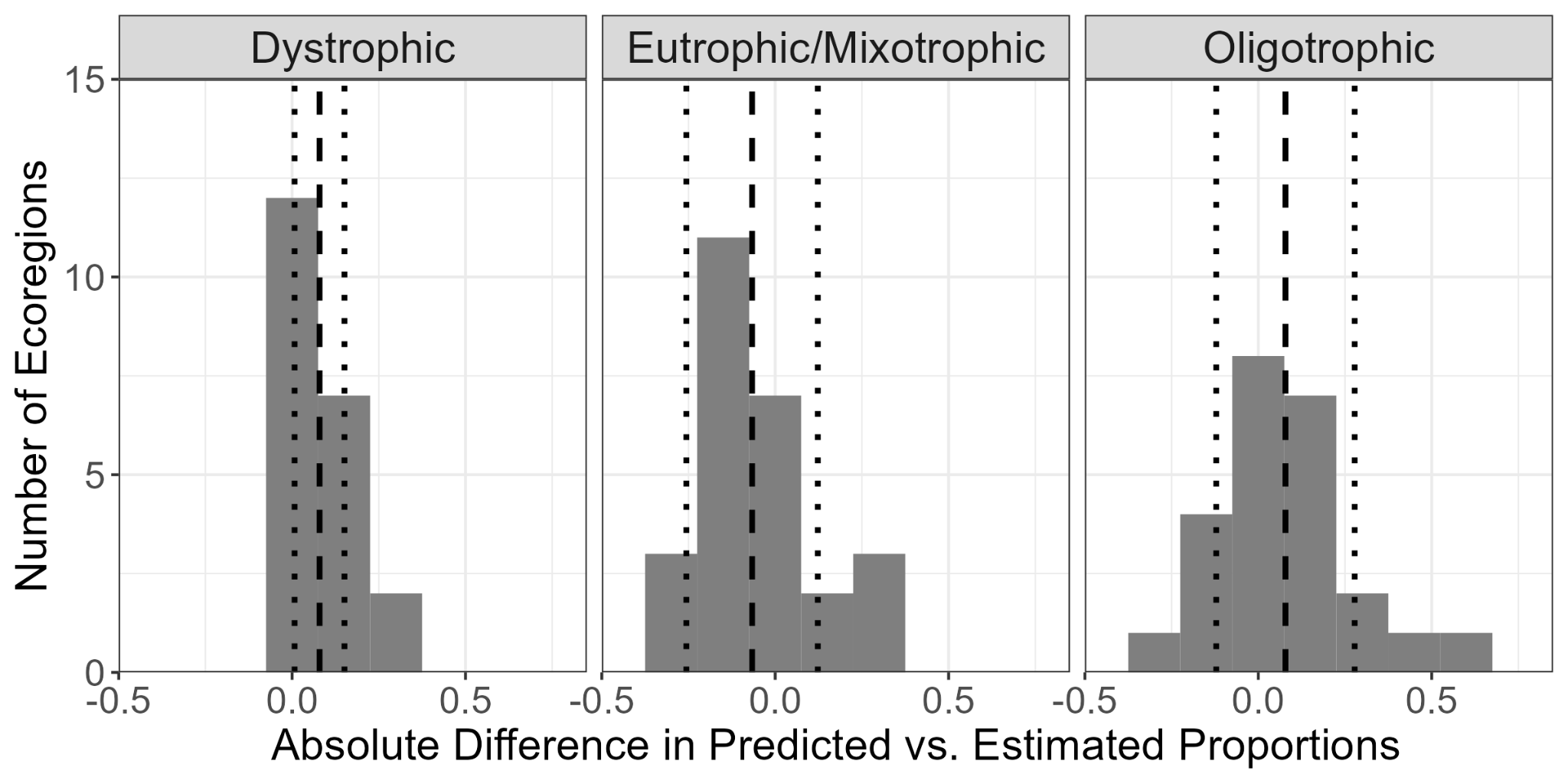


Figure S24: Histograms of absolute difference (Estimated - Predicted) in predicted and estimated proportions of each lake trophic state across U.S. EPA Level I Ecoregions. Vertical dashed lines reflect the mean, and vertical, dotted lines reflect one standard deviation from the mean. For all trophic states, distributions approximately center around zero. Oligotrophic and dystrophic lakes tend to be slightly underpredicted, whereas eutrophic and mixotrophic lakes tend to be slightly overpredicted.


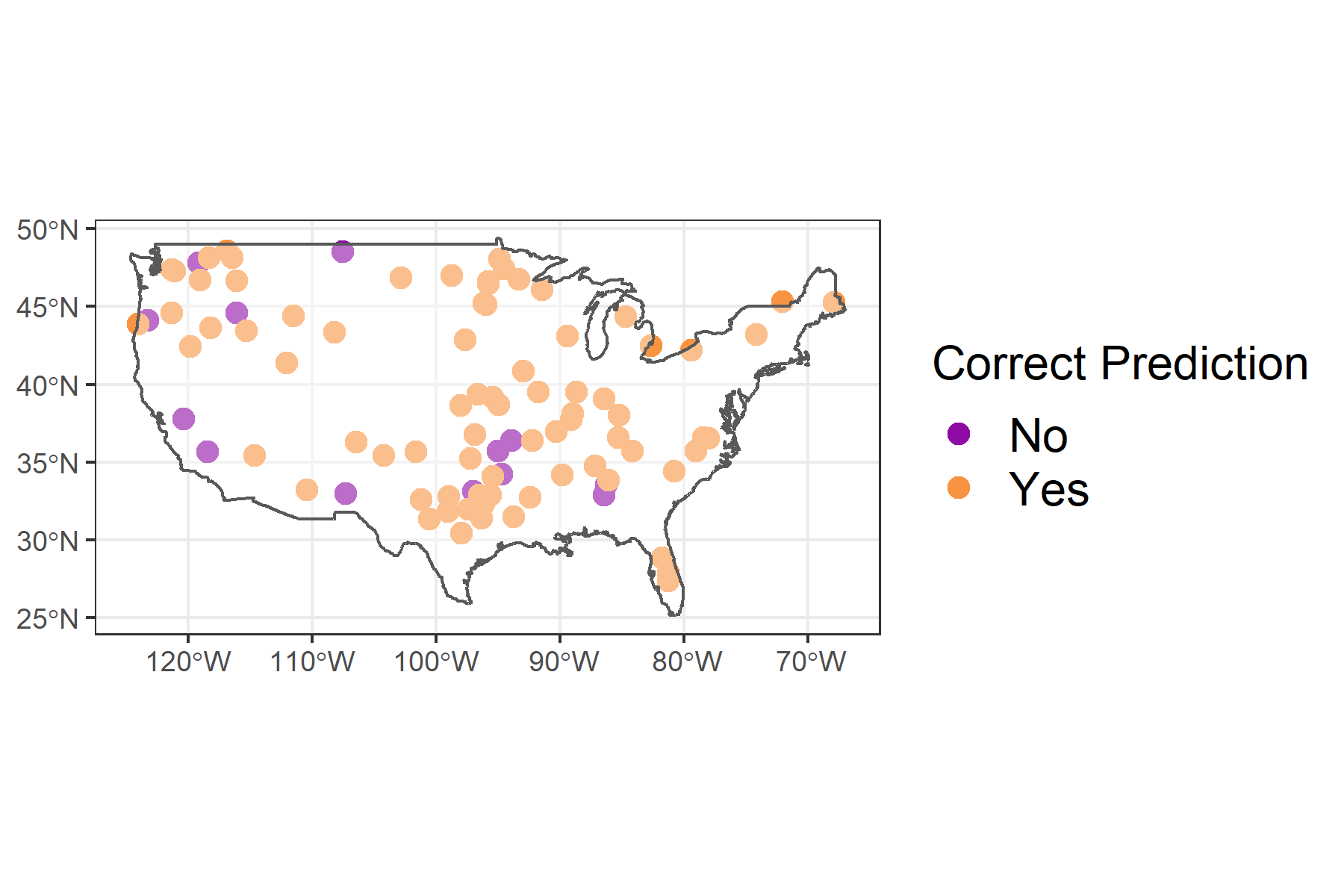


Figure S25: National-scale map of correct and incorrect trophic state classifications as assessed by manual checking of lake trophic state predictions against independent sources. Among lakes where independent sources could be identified, 73.5% of lakes were correctly predicted, which is notably similar to accuracies assessed from the NLA sampling campaign data. Additionally, correct and incorrect classifications did not follow apparent spatial patterns, implying that our models were not influenced by geographical or locational differences.
